# The Light Intermediate Chain 2 Subpopulation of Dynein Regulates Mitotic Spindle Orientation

**DOI:** 10.1038/s41598-016-0030-3

**Published:** 2016-12-23

**Authors:** Sagar Mahale, Megha Kumar, Amit Sharma, Aswini Babu, Shashi Ranjan, Chetana Sachidanandan, Sivaram V. S. Mylavarapu

**Affiliations:** 1 Laboratory of Cellular Dynamics, Regional Centre for Biotechnology, NCR Biotech Science Cluster, 3rd Milestone, Faridabad-Gurgaon Expressway, Faridabad, Haryana 121001 India; 2 Affiliated to Manipal University, Manipal, Karnataka 576104 India; 3 CSIR-Institute of Genomics & Integrative Biology, South Campus, New Delhi, 110025 India; 4 Academy of Scientific and Innovative Research (AcSIR), New Delhi, 110025 India

## Abstract

Cytoplasmic dynein 1 is a multi-protein intracellular motor essential for mediating several mitotic functions, including the establishment of proper spindle orientation. The functional relevance and mechanistic distinctions between two discrete dynein subpopulations distinguished only by Light Intermediate Chain (LIC) homologues, LIC1 and LIC2 is unknown during mitosis. Here, we identify LIC2-dynein as the major mediator of proper spindle orientation and uncover its underlying molecular mechanism. Cortically localized dynein, essential for maintaining correct spindle orientation, consists majorly of LIC2-dynein, which interacts with cortical 14-3-3 ε- ζ and Par3, conserved proteins required for orienting the spindle. LIC2-dynein is also responsible for the majority of dynein-mediated asymmetric poleward transport of NuMA, helping focus microtubule minus ends. In addition, LIC2-dynein dominates in equatorially aligning chromosomes at metaphase and in regulating mitotic spindle length. Key mitotic functions of LIC2 were remarkably conserved in and essential for early embryonic divisions and development in zebrafish. Thus LIC2-dynein exclusively engages with two major cortical pathways to govern spindle orientation. Overall, we identify a novel selectivity of molecular interactions between the two LICs in mitosis as the underlying basis for their uneven distribution of labour in ensuring proper spindle orientation.

## Introduction

Somatic metazoan cells divide via the process of mitosis with high fidelity to generate two daughter cells that contain the correct complement of chromosomes. The ubiquitous molecular motor cytoplasmic dynein 1 (referred to henceforth as “dynein”) mediates several important mitotic processes^1–5^. These include chromosome congression to the cell equator^6–8^, regulation of mitotic spindle assembly, length and spindle pole focusing^3, 9–12^, astral microtubule nucleation from spindle poles^13^, proper spindle positioning^14, 15^ and establishment of correct spindle orientation^16, 17^.

Dynein is a large, non-covalent cytoplasmic complex of protein subunits including heavy chains, intermediate chains, light intermediate chains and light chains^1, 2, 18–20^. Of these, the Light Intermediate Chains (LICs) have been poorly studied in the context of mitosis. Invertebrates express only a single LIC^21–24^, while vertebrates have evolved to express two distinct LIC homologues, LIC1 and LIC2, which occupy cytoplasmic dynein 1 in mutually exclusive dynein complexes^25^. Recent studies have illuminated the biochemical basis for assembly of LICs into the dynein complex and suggested cargo-binding functions for the LICs, and also illuminated their role in spindle pole focusing^12, 26, 27^. A thorough functional and mechanistic dissection of the two LICs during mitosis is however missing. This knowledge is required to understand their relative contributions in mitosis as well as to discern the evolutionary significance of the emergence of two LICs in vertebrates.

Here, we show that the LIC2 fraction of cytoplasmic dynein plays a dominant role in orienting the mitotic spindle. LIC2 depletion in cells resulted in prolonged arrest in mitosis and was characterized by severe spindle mis-orientation as compared to LIC1 depletion. LIC2-dynein, but not LIC1-dynein interacts with Par3 and the 14-3-3 ε and ζ proteins, which constitute key components of the spindle orientation apparatus in mammalian cells^14, 28, 29^. Mis-oriented spindles in LIC2-depleted cells showed reduced amounts of NuMA at the defectively anchored spindle pole and a concomitant accumulation at the corresponding cortex. In addition, LIC2 also plays a dominant role in ensuring chromosome congression at the cell equator during metaphase, while LIC1 plays a larger role in maintaining spindle pole integrity. The LICs showed overlapping roles in cultured cells and zebrafish embryonic divisions, suggesting that the role of LICs in vertebrate development is evolutionarily conserved. Overall, our study uncovers an unequal distribution of mitotic functions between the two LIC-containing dynein complexes in vertebrates and reveals the molecular mechanisms by which LIC2-dynein dominates to ensure proper spindle orientation.

## Results

### LIC2 is required for mitotic progression

**Figure 1:**
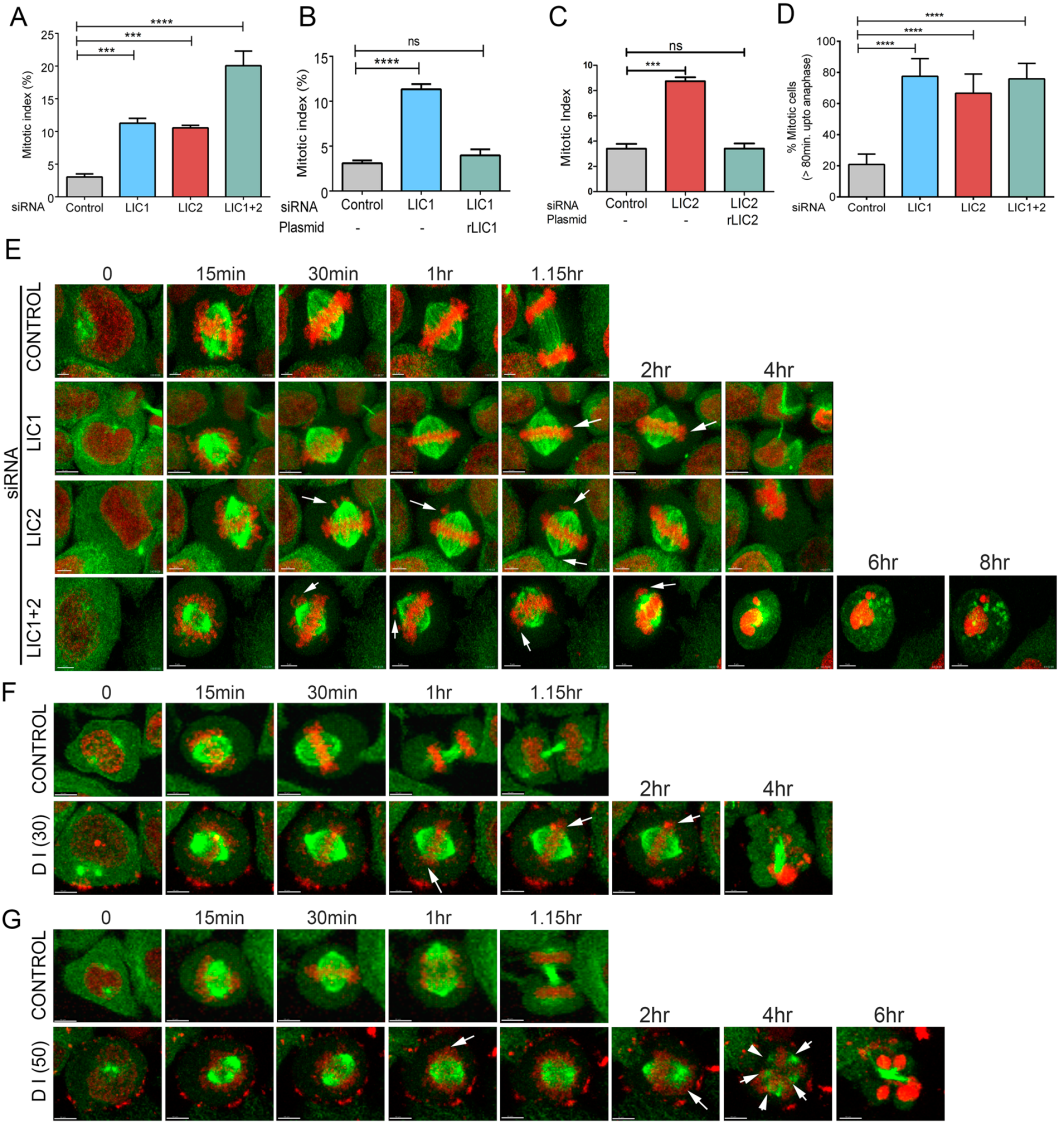
LIC2 is required for mitotic progression in HeLa cells. (**A**) Mitotic index in HeLa cells (y-axis); the % of total cells present in metaphase +/−SD. (3 experiments, n = approximately 500 cells per experiment). (**B**,**C**) Rescue of the metaphase arrest by transgenic expression of rat LIC1 (**B**) and rat LIC2 (**C**) (3 experiments, n = at least 500 cells per experiment). (**D**) Fraction of mitotic cells from G that spent >80 minutes in mitosis. p values in all panels are *p < 0.05, **p < 0.01, ***p < 0.001. (**E**) Still images from movies featuring different siRNA treated HeLa cells expressing H2B-mCherry and GFP-α-tubulin (control, LIC1, LIC2 and LIC 1 + 2 depleted) showing prolonged arrest in mitosis. Time points indicate time after nuclear envelope breakdown. (**F**) Still images from movies representing effect of dynein inhibitor ciliobrevin (DI) at different concentrations 30 μM (**F**) and 50 μM (**G**) with DMSO as control.

Between the two vertebrate LIC homologues, the mitotic functions of LIC2 remain less understood. A role for LIC2 in mediating completion of cytokinesis had been reported^30^. Recently, dynein LICs were reported to have roles in mitosis^4^ and in maintaining spindle bipolarity^12^. LIC2 and LIC1 both prominently decorated mitotic centrosomes, spindle microtubules and the spindle midzone in anaphase^31, 32^. We confirmed the specificity of the antibodies used for immunofluorescence by depleting either LIC1 or LIC2 alone, using sequence specific siRNAs (Supplementary Fig. S1A–C ^30, 32^). The clear and reproducible arrest in mitosis upon LIC2 depletion (Fig. 1A ^4^) was independent of LIC1 depletion (Fig. 1A, Supplementary Figs S1A–C and S2A,B), despite using siRNAs of similar potency (Supplementary Fig. S1D). Only co-depletion of both LICs by treatment with a mixture of both siRNAs led to an additive mitotic arrest (Fig. 1A), suggesting that LIC1 and LIC2 facilitate mitotic progression by largely independent mechanisms. The mitotic arrest could be rescued by exogenous expression of the corresponding rat LIC orthologs that were not targeted by the respective anti-human LIC siRNAs (Fig. 1B,C), further verifying the specificity of the depletion phenotype. The mitotic arrest upon LIC2 depletion was also observed in retinal pigment epithelial (hTERT-RPE1) cells and could be specifically rescued by transgenic expression of rat LIC2 (Supplementary Fig. S1E–G). Time-lapse video imaging (Fig. 1E) showed that LIC1, LIC2 and LIC1-LIC2 co-depleted cells took significantly longer to proceed from nuclear envelope breakdown (NEB) to anaphase (Supplementary movies 2, 3 and 4 respectively) as compared to control cells (Supplementary movie 1), confirming the mitotic arrest (Fig. 1D). Treatment with the dynein inhibitor ciliobrevin D as a positive control led to prolonged mitotic arrest (Fig. 1F,G, Supplementary movies 6 and 8) followed by cell death, as compared to normal mitotic progression in control DMSO (solvent) treated cells (Fig. 1F,G, Supplementary Fig. S2C and movies 5 and 7).

**Figure 2:**
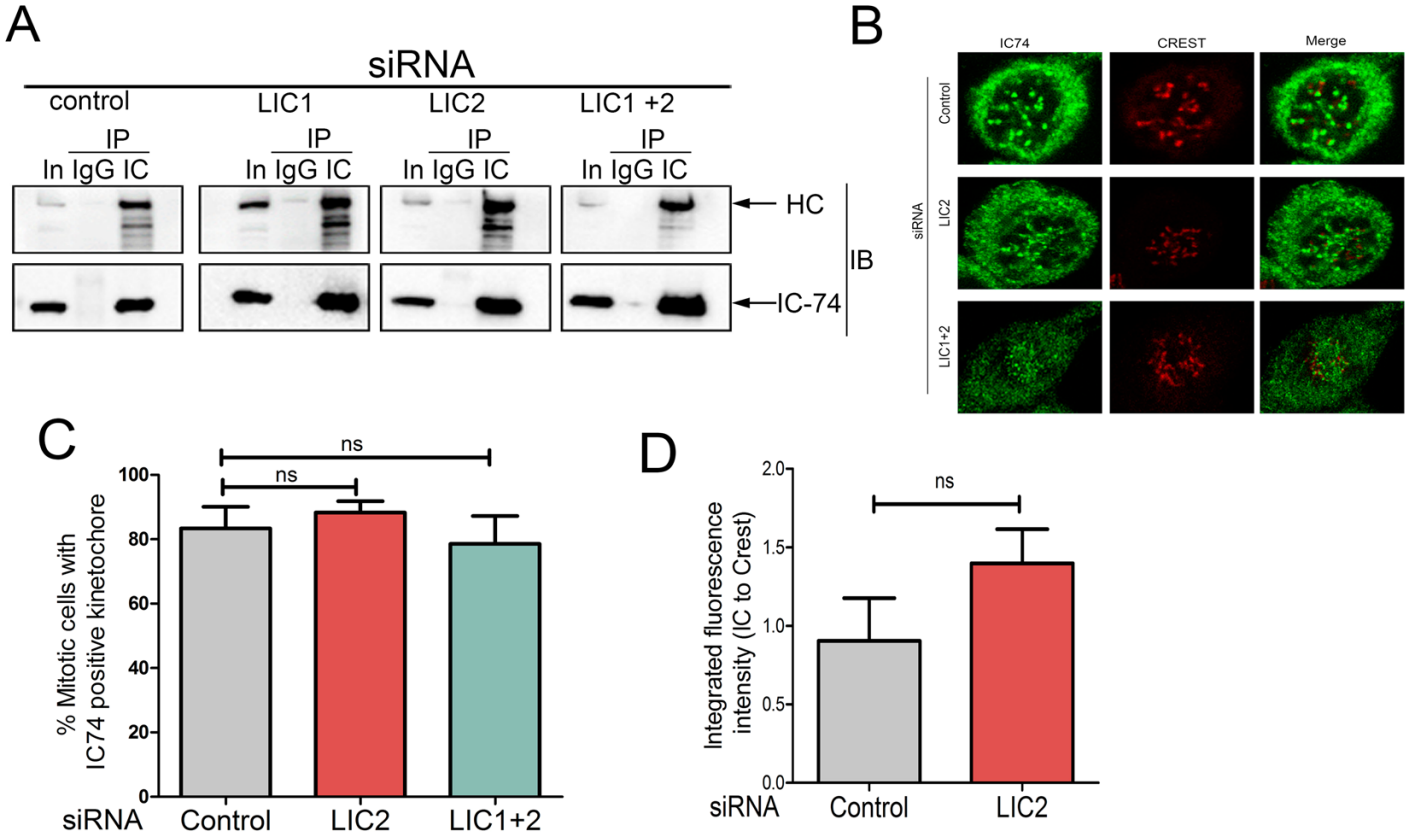
Dynein retains its integrity and mitotic localization upon LIC2 depletion. (**A**) Immunoprecipitation with IC74 co-precipitates HC irrespective of LIC depletion. In = Input, IP = immunoprecipitation, IB = immunoblot. (**B**) Representative confocal immunofluorescence images showing localization of IC74 to kinetochores (CREST). (**C**) Percent HeLa cells showing the proper localization of IC74 to kinetochores (CREST) upon respective siRNA treatment. (3 experiments, n = at least 17 prometaphase cells per experiment). (**D**) Integrated fluorescence intensity of IC74 staining as a ratio to CREST staining at kinetochores from the cells in C (10 cells per experiment from 3 experiments).

We sought to ascertain the integrity of the dynein complex upon depletion of LIC2^18, 19^. We performed immunoprecipitation assays with the intermediate chain (IC74), a core dynein subunit^2, 18, 19, 33^ in control siRNA treated cells, LIC2-depleted cells and cells co-depleted of LIC1 and LIC2. We immunoblotted the precipitates for dynein heavy chain (HC), the scaffold of the dynein complex, to probe for interaction of HC and IC74 in the same complex. The HC was pulled down with IC74 irrespective of LIC depletion (Fig. 2A), however we see the remnant LICs preferentially pulled down in the IC74 IPs upon LIC depletion as well, albeit with markedly reduced levels of LICs as compared to control (Supplementary Fig. S10). This suggested that the majority of HC and IC formed reasonably stable complexes in cells despite the absence of the LICs, consistent with multiple previous studies that show co-sedimentation of the HC (~500 kD) and IC (~74 kD) in the same density gradient fractions irrespective of LIC knockdown^12, 30, 33^. The LICs seem to be important for HC dimerization and stability *in vitro* as shown by reconstitution studies^34, 35^. It is possible that the presence of additional binding partners in the cell lysate could serve to partially stabilize the HC-IC interaction even in the absence of the LICs, while complete stability is achieved in the presence of all subunits in the correct stoichiometric ratios. IC74 also retained its normal mitotic localization at prometaphase kinetochores in a manner indistinguishable from control cells (Fig. 2B,C). Fluorescence quantification showed that there was no loss of kinetochore IC74 levels upon LIC2 depletion (Fig. 2D). These results show that the biochemical integrity and localization of cytoplasmic dynein were normal upon LIC2 depletion, consistent with existing literature^4, 12, 30, 33^.

### LIC2 governs mitotic spindle orientation

**Figure 3:**
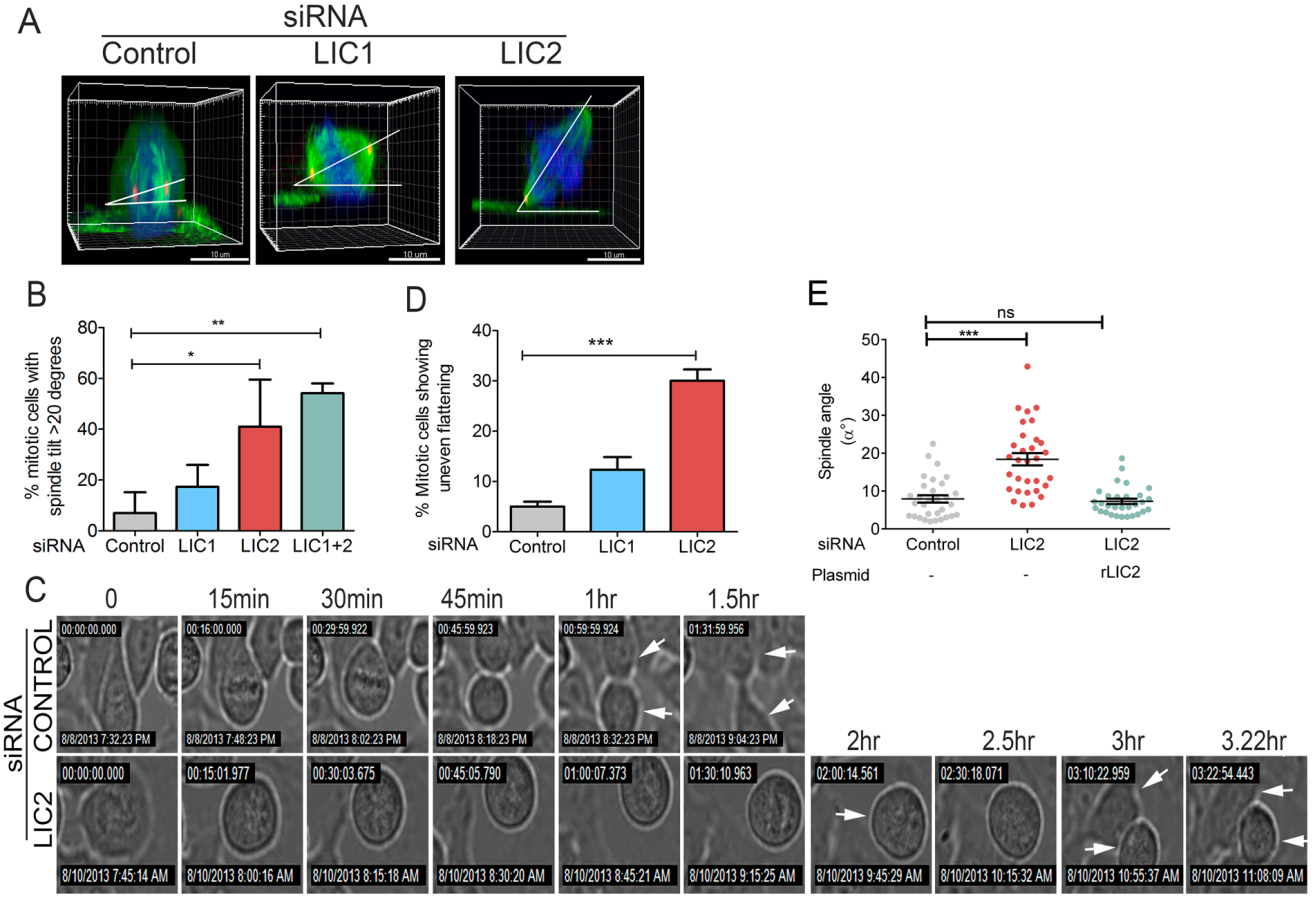
LIC2 regulates spindle orientation in mitosis. (**A**) Spindle angle in representative mitotic HeLa cells depicted from 3D reconstructions of confocal z-stacks, after respective siRNA treatment. (**B**) Fraction of metaphase cells with a spindle angle of >20 degrees. Error bars = +/− SEM from 3 independent experiments, at least 20 metaphase cells per experiment. (**C**) Still images from time-lapse movies of HeLa cells exiting mitosis showing uneven post-mitotic flattening upon LIC2 depletion at various time points after rounding up; arrows indicate daughter cells. (**D**) Percent mitotic cells showing uneven flattening (3 experiments, n = minimum 10 mitotic cells per experiment). Error bars are mean +/− SD from 3 independent experiments. (**E**) Rescue of the spindle orientation defects by transgenic expression of rat LIC2. Y-axis represents the spindle angle with respect to the substratum (at least 30 cells over 3 experiments, Error bars are mean +/− SEM, p values in all panels are *p < 0.05, **p < 0.01, ***p < 0.001).

The predominant mitotic localization of the LICs at spindle poles suggested that they may contribute to spindle orientation, an important spindle pole-related function of cytoplasmic dynein^13, 14, 36–38^. HeLa cells treated with LIC-specific siRNAs were visualized by time-lapse imaging on gridded cover slips to identify cells that arrested for prolonged periods (between 80 minutes to 4 hours from NEB) in mitosis to identify LIC-depleted cells. Cells arrested for longer than 4 hours in mitosis often died^32^. We observed that LIC2 depletion led to drastic spindle mis-orientation (Fig. 3A) as compared to control cells, with over 40% metaphase cells showing a spindle tilt of greater than 20 degrees with respect to the substratum (Fig. 3B and Supplementary movies 9–control and 10–LIC2 depletion). In contrast, LIC1 depletion showed minimal spindle mis-orientation (Fig. 3A,B), suggesting a stronger role for LIC2-dynein. In order to ascertain the contribution of adhesion to the substratum, if any, on this phenotype, we performed a similar experiment on collagen-coated cover slips to engage the integrins and ensure proper adhesion^39^. We observed a similar trend, with pronounced spindle mis-orientation seen specifically only upon LIC2 depletion, (Supplementary Fig. S3A–D). LIC2-depleted cells also showed distinctly uneven flattening of daughter cells after anaphase in comparison to control cells that divided parallel to the substratum and thus flattened evenly (Fig. 3C). This result demonstrated a substantial tilt in the orientation of the division plane (Fig. 3D and Supplementary movies 11–control and 12–LIC2 depletion). The orientation defects were rescued by exogenous expression of the corresponding rat LIC2 ortholog that was not targeted by the anti-human LIC2 siRNA (Fig. 3E), further verifying the specificity of the LIC2 depletion phenotype. We also checked whether the prolonged arrest in mitosis upon LIC depletion itself might have caused spindle mis-orientation, by arresting cells in metaphase for prolonged periods upon treatment with the proteasome inhibitor MG132. We observed that prolonged arrest up to 4 hours by itself did not cause significant spindle mis-orientation in control cells^39, 40^ and LIC1 depleted cells, but rather only upon LIC2 depletion (Supplementary Fig. S4A,B and Supplementary movies 13, 14 and 15). These results together show a dominant contribution of LIC2-dynein in maintaining proper mitotic spindle orientation.

### LIC2-dynein, but not LIC1-dynein transports NuMA asymmetrically to the spindle poles

We next aimed to delineate the molecular mechanism(s) by which LIC2 regulates spindle orientation. NuMA (Nuclear Mitotic Apparatus), a large conserved nuclear protein has been clearly implicated in focusing microtubule minus ends at spindle poles^41, 42^. Both NuMA and dynein are also key components of a crucial cortical protein complex responsible for capturing the plus ends of astral microtubules, thus anchoring spindle poles to the cortex, thereby achieving spindle orientation^14–16, 36, 43–47^. It has also been reported that NuMA is transported by dynein along microtubules to the poles, although this interaction is weak^16, 37, 43, 45, 48^.

**Figure 4:**
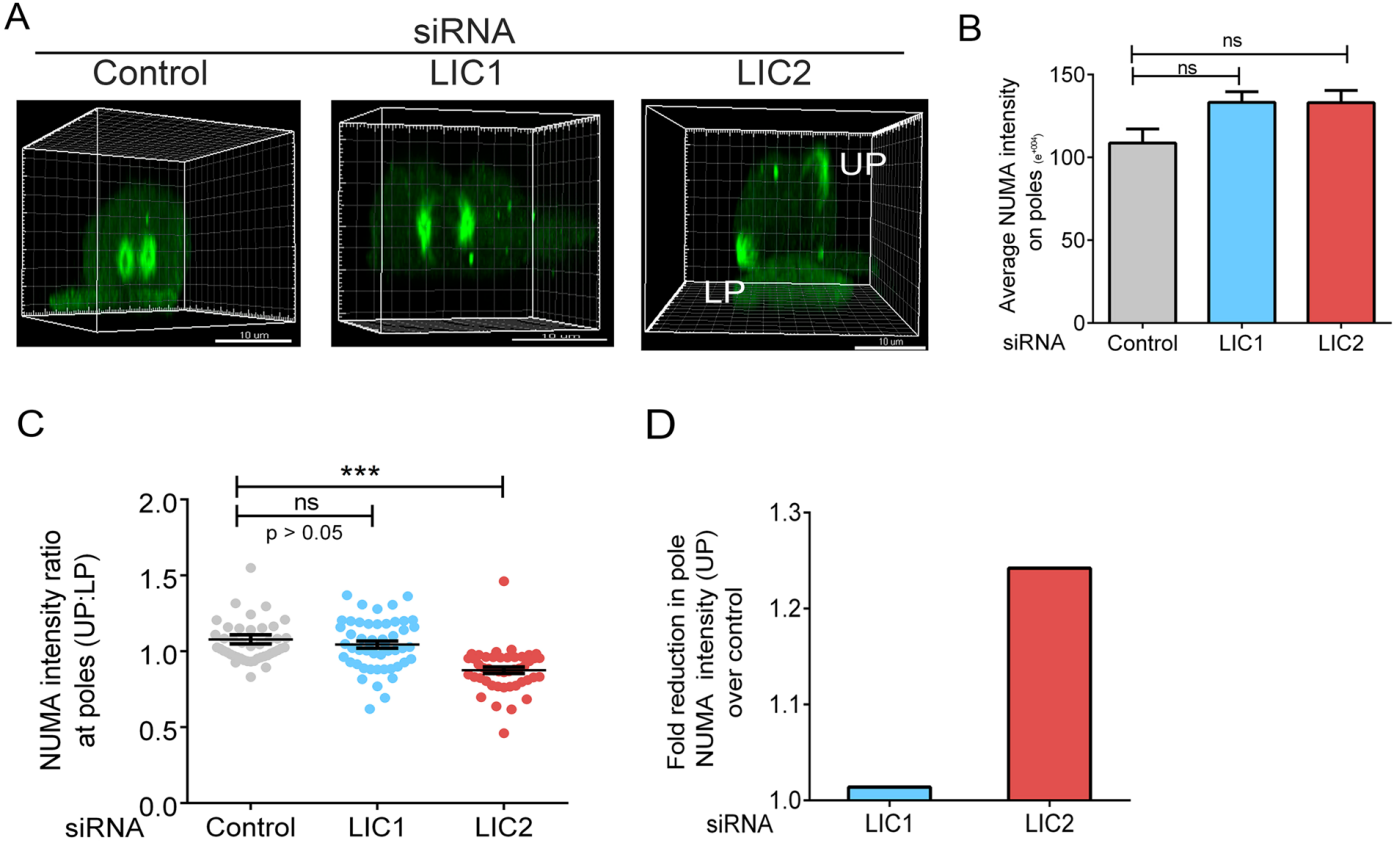
LIC2 transports NuMA to the spindle pole during mitosis. (**A**) Representative 3D reconstructions from confocal immunofluorescence images showing spindle poles in mitotic HeLa cells. LP = lower pole, UP = upper pole in LIC2 depleted (mis-oriented) cells. (**B**) Average NuMA intensity at poles from quantification using 3-dimensional regions of interest drawn around the centrosomes. Error bars are mean +/− SEM from 3 independent experiments, at least 20 metaphase cells per experiment. (**C**) Ratio of fluorescence intensities (UP/LP) in metaphase cells–54 cells (control), 57 cells (LIC1 depleted) and 55 cells (LIC2 depleted) over a total of 3 experiments. Error bars are mean +/− SEM from 3 independent experiments. (**D**) Fold reduction in average NuMA levels at the upper pole upon LIC2 depletion in comparison to control cells from the data used for C.

We probed whether LIC2-dynein preferentially influenced intracellular NuMA localization, since the prominent localization of LIC2 to mitotic spindle poles^32^ mirrored the polar localization of NuMA. We could distinctly observe the organization of NuMA into well-defined, ring-like structures around mitotic centrosomes in control cells (Fig. 4A). Upon LIC2 depletion, the “upper” (farther from substratum) poles of mis-oriented cells had less NuMA intensity as compared to the lower poles (Fig. 4A), which we quantified using 3D reconstructed images (Fig. 4B–D). When all centrosomes were analyzed, depletion of LIC2 did not appear to significantly affect the amounts of polar NuMA immunofluorescence with respect to control cells (Fig. 4B), consistent with a recent report^12^. However, careful analysis of the 3D quantification of spindle poles revealed that there was a small but significant reduction (by 15–20%) of NuMA intensity preferentially at the “upper” spindle pole upon depleting LIC2 but not LIC1 (Fig. 4C–F). This result indicated that LIC2-dynein is responsible for mitotic transport of NuMA preferentially to one spindle pole. We observed spindle pole focusing defects upon LIC2 depletion (Supplementary Fig. S8 **)**, which can partially be attributed to the defective transport of NuMA to the pole, in addition to the role of LICs in centriole cohesion as recently reported^12^. The strong spindle orientation defects observed upon LIC2 depletion prompted us to examine whether there was any change in NuMA localization at the corresponding polar cortex, since cortical NuMA is crucial for ensuring proper spindle orientation. Analysis of linescan intensities as well as region of interest (ROI) measurements^36^ revealed a significant accumulation of NuMA at the upper cortex only (corresponding to the upper pole) upon depletion of LIC2 but not of LIC1 (Supplementary Figs S5A–F and S6). The fold NuMA accumulation at the upper cortex upon LIC2 depletion (Supplementary Fig. S5E) was similar to the fold reduction of NuMA at the upper pole (Fig. 4D). Thus, we conclude that NuMA is transported preferentially to one spindle pole from the cortex and that this transport is mediated primarily by LIC2-dynein, but not by LIC1-dynein.

### LIC2-dynein exclusively interacts with key protein complexes that govern spindle orientation

**Figure 5:**
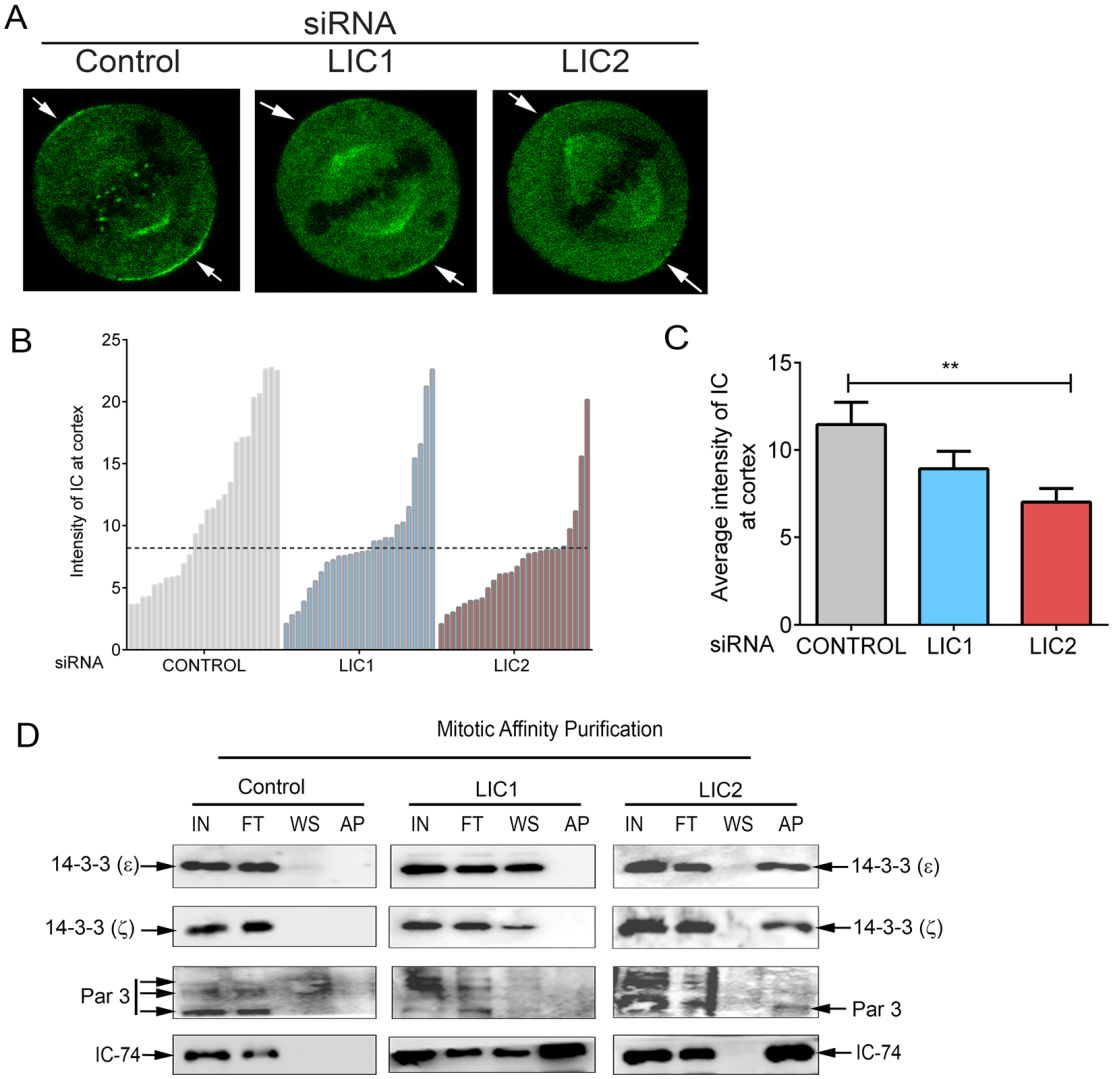
LIC2-dynein interacts with cortical orientation complexes. (**A**) Still images from time-lapse movies of HeLa cell line expressing multifunctional GFP-tagged IC74 (mfGFP-IC74). Arrows indicate cortical dynein. (**B**) Quantification of cortical intensity of mfGFP-IC74 upon respective siRNA treatments. Y-axis represents average fluorescence intensity at the cortex, each bar represents one cell. Horizontal dashed line marks the average fluorescence intensity seen in LIC2-depleted cells. N = 26 cells each for all conditions. LIC2-depleted cells show significantly reduced mfGFP-IC74 cortical intensity in comparison to control or LIC1-depleted cells. (**C**) Quantification of mfGFP-IC74 intensity at the cortex upon respective siRNA treatment. Bars represent mean +/− SEM from 3 independent experiments. N = 26 live metaphase cells from 3 independent experiments). (**D**) Affinity purification of SBP-tagged empty vector (control), LIC1 and LIC2 followed by immunoblotting with respective antibodies as indicated. IN = input, FT = flow through, WS = wash, AP = affinity purified eluate.

Cortical NuMA serves as a major recruiting factor for cortical dynein. We next examined whether LIC1- or LIC2-dynein levels were predominant at the cortex. Using a multifunctional GFP-tagged IC74 Hela cell line (mfGFP-IC74 Hela^49^), we could visualize the dynamic localization of mitotic dynein on the mitotic spindle, the spindle poles, the kinetochores and at the pole-proximal cortex (Fig. 5A, Supplementary movie 16 and Supplementary Fig. S7). Quantification of mfGFP-IC74 fluorescence intensity at the cortex from stills of these movies using published methods^36^ showed that the levels of cortical dynein dropped significantly upon LIC2 depletion (Supplementary movie 18 and Supplementary Fig. S7). A substantial fraction of LIC1-depleted cells showed normal dynein (mfGFP-IC74) levels at the cortex (Supplementary movie 17 and Supplementary Fig. S7), while all LIC2 depleted cells analyzed showed reduced cortical IC74 levels (Fig. 5B). Quantitative comparison of fluorescence intensities revealed significant reduction in mfGFP-IC74 levels at the cortex upon LIC2 depletion, but a minor reduction upon LIC1 depletion (Fig. 5C). The above results together suggest that LIC2-dynein transports NuMA from the cortex to the spindle pole during mitosis.

The other major cortically localized protein complex responsible for maintaining proper spindle orientation is the Par3-aPKC complex^28, 29, 50^. Interestingly, Par3 interacts specifically with LIC2 but not with LIC1 in interphase cells, helping to position the centrosome^51^. Given that we observed strong spindle orientation defects upon LIC2 depletion (Fig. 3), we probed whether the specific interaction of Par3 with LIC2 is also observed during mitosis. We used mitotically enriched lysates from cells stably expressing affinity tagged (Streptavidin Binding Protein or SBP-tagged) LIC1 or LIC2 to probe for the interaction of Par3. Affinity purification eluates from mitotically enriched lysates were analyzed for the presence of Par3 by immunoblotting. Indeed, we observed Par3 (~100 kD isoform) in the affinity pull-downs of LIC2 but not of LIC1 or control affinity tag alone (Fig. 5D). This result suggested that the exclusive LIC2-Par3 interaction also exists during mitosis and contributes to the spindle orientation functions of LIC2-dynein. We also probed for the possible interaction of the LICs with the 14-3-3 proteins ε and ζ in the affinity purification eluates, since these proteins are known to interact with cytoplasmic dynein^28, 52^ and to biochemically link the Par3 and NuMA spindle orientation pathways to achieve complete spindle orientation^28^. We observed robust interaction of both 14-3-3 ε and ζ proteins with LIC2, but no interaction with either LIC1 or with the control empty tag alone (Fig. 5D). Together, the above results revealed exclusive engagement of LIC2-dynein, but not of LIC1-dynein with both the NuMA and Par3 containing cortical protein complexes that collaborate to achieve proper spindle orientation.

### LIC2-dynein and LIC1-dynein distribute other mitotic functions

Cytoplasmic dynein facilitates chromosome congression to the metaphase plate^53^. As described above, we identified cells that had arrested in metaphase for prolonged intervals upon LIC2 depletion and examined them by immunofluorescence imaging of kinetochores, chromosomes and spindle poles. LIC2-dynein played a major role in ensuring proper chromosome congression at the cell equator. Over 50% of LIC2 depleted cells that had arrested in metaphase for prolonged periods showed incompletely congressed metaphase plates, an approximately 3-fold defect in chromosome congression in comparison to control cells. LIC1 played a comparatively minor role in chromosome congression (Supplementary Fig. S9A,B). We also observed that mitotic spindles were significantly elongated in LIC2 depleted cells, with the spindle poles almost juxtaposed with the cell cortex, an effect much less pronounced for LIC1 depletion (Supplementary Fig. S9C,D). This observation suggested a dominant role for LIC2-dynein in regulating spindle length. The elongated spindle length is likely due to the loss of dynein function in shortening the spindle^17, 54, 55^. As opposed to these phenotypes, LIC1-dynein was the dominant dynein fraction required to prevent major spindle pole fragmentation. LIC1-depleted cells showed a higher fraction of cells with gamma-tubulin positive foci scattered in the cytoplasm (Supplementary Fig. S8). To test whether pole fragmentation was a consequence of prolonged mitotic arrest alone^40^, we arrested cells in metaphase for up to 4 hours using treatment with MG132. The fragmentation phenotype was consistently observed only upon LIC1 depletion (Supplementary Fig. S4C,D, Supplementary movies 19, 20 and 21). These observations collectively suggested a strong involvement for LIC2 in regulating the length of the mitotic spindle as well as congressing chromosomes to the equatorial plate. LIC1 makes only minor or negligible contributions in these functions, but plays a larger role in maintaining spindle pole integrity.

### LIC2 is required for early vertebrate embryonic divisions and development

**Figure 6:**
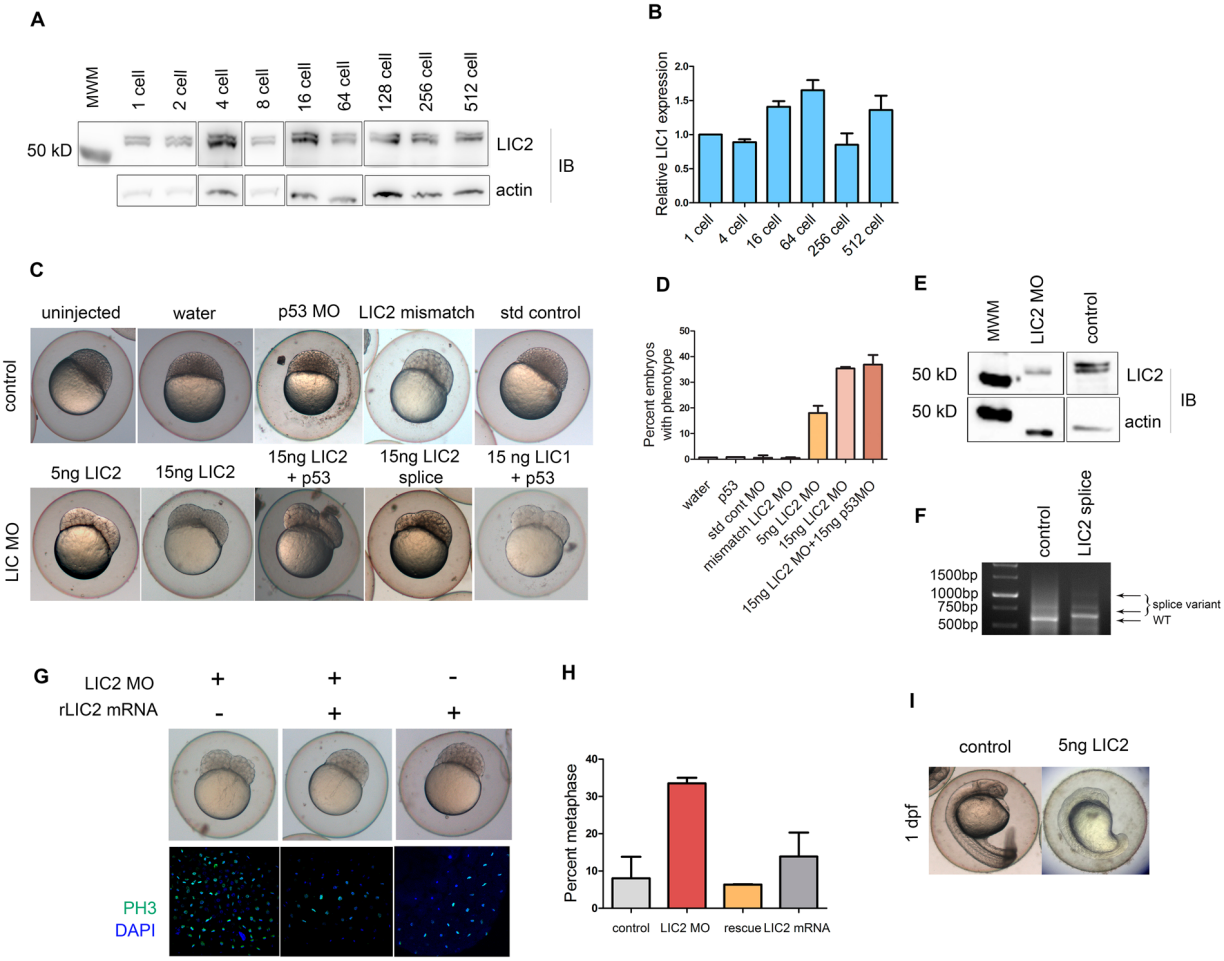
Zebrafish LIC2 is required for early embryonic development. (**A**) zLIC2 expression at early stages of zebrafish embryogenesis, as shown by immunoblotting of whole embryo lysates. (**B**) zLIC1 expression at early stages of zebrafish embryogenesis, as shown by real time PCR analysis. (**C**) Knockdown of zLIC2 in zebrafish embryos. Top–control embryos as indicated. Below–depletion of zLIC2 by zLIC2-specific morpholinos as indicated. (**D**) Fraction of embryos showing the furrow phenotype. (**E**) Western blot showing zLIC2 knockdown using translation blocker LIC2 MO (the top zLIC2 band completely disappears upon MO treatment). *β*-actin serves as loading control. (**F**) Reverse transcriptase PCR showing splice variants in LIC2 splice morpholino injected embryos (arrows). Lane 1 = molecular weight ladder. (**G**) Gross embryo morphology (top) and phosphohistone 3 staining (bottom) of embryos injected with LIC2 morpholino and/or rLIC2 mRNA as indicated. (**H**) Fraction of cells in metaphase from morphants, rescued and mRNA injected embryos from G. N = 17, 28 and 20 embryos respectively from 2 experiments. (**I**) Representative embryo injected with 5 ng translation blocker MO against zLIC2 show developmental delays at 1 dpf.

The role of dynein during early embryonic development is not well understood. We probed the function of LIC2 in controlling early divisions in the zebrafish embryo. Immunoblotting and real time PCR analysis revealed that both zebrafish LICs were expressed across early embryonic stages up to the 512-cell stage, suggesting maternally inherited expression (Fig. 6A,B). We depleted zLICs from zebrafish embryos by injecting sequence-specific morpholinos into one-cell embryos. zLIC2 depleted embryos (using both translation-blocking and splice-blocking morpholinos specific to zLIC2) exhibited a distinct “furrow” phenotype at the blastula stage (around the 256–512 cell stage, Fig. 6C). The blastomeres of zLIC2 morphant embryos segregated into two distinct “hemispheres”, with a central furrow separating them. Control morpholino injected embryos (injected with water, p53, standard control or LIC2 mismatch morpholinos) showed normal embryonic development indistinguishable from uninjected embryos (Fig. 6C). The intensity of the furrow phenotype varied in a dose-dependent manner with the amount of LIC2 morpholino injected (Fig. 6C,D). We co-injected p53 morpholino to reduce non-specific, off-target effects of the morpholinos^56^. Immunoblotting of whole-embryo lysates obtained from furrowed embryos showed a specific decline in the levels of LIC2 protein (Fig. 6E). We also verified specificity of the knockdown by injecting a splice blocking morpholino against zLIC2, which resulted in alternatively spliced zLIC2 variants (Fig. 6F). We further confirmed the specificity of the zLIC2 depletion phenotypes by performing functional rescue experiments by introducing an orthologous rat LIC2 mRNA, which ameliorated the gross furrow phenotype in almost all treated embryos (Fig. 6G, top). LIC2 depleted embryos showed an increased fraction of cells in metaphase by phosphohistone 3 (PH3) staining (6G bottom, 6H) suggesting an arrest in cell division, which was reversed upon rescue with rat LIC2 mRNA (Fig. 6G,H). Furrowed embryos failed to develop further; however embryos injected with a lower dose of morpholino exhibited slower development around 1 day post fertilization (1 dpf), with poorly formed head and tailbud and defects in elongation of the body axis (Fig. 6I). The above results demonstrated that LIC2 is required for early development in zebrafish embryos.

**Figure 7:**
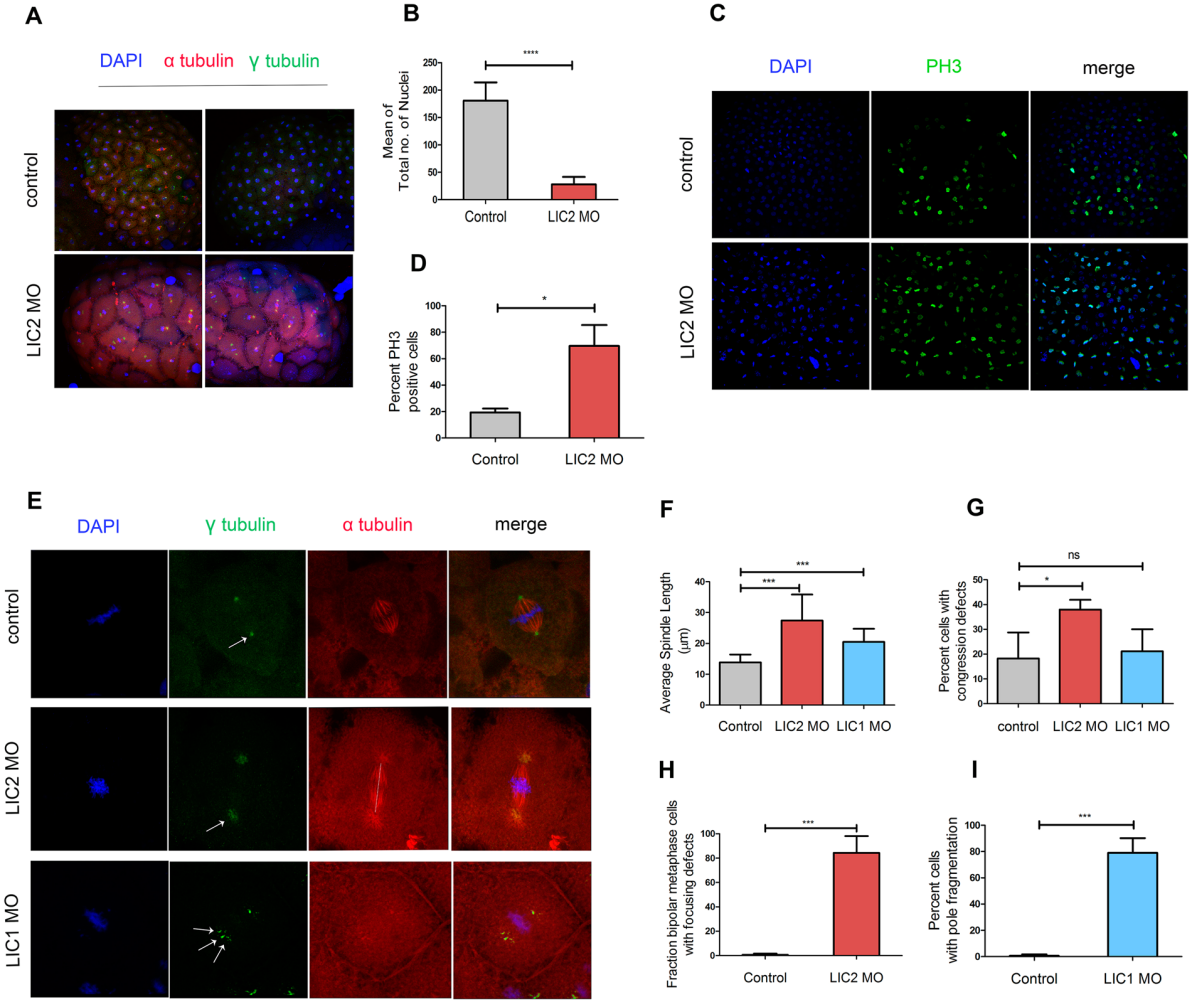
Zebrafish LIC2 depletion leads to mitotic defects in early embryonic divisions. (**A**) Cytology of blastomeres upon LIC2 depletion. Each representative image shows the top surface view of the embryo at 3.3 hpf, recreated from confocal z-stacks. Control = embryos injected with standard control MO. Embryos are stained for microtubules (red), centrosomes (green) and chromosomes (blue). (**B**) Total average number of nuclei per embryo in surface blastomeres of time-matched embryos (minimum 3 experiments, n = 10 control and 23 LIC2 depleted embryos). (**C**) Sum projections of confocal z stacks showing mitotic cells labelled with phosphohistone 3 (PH3, green) and chromatin (DAPI, blue). (**D**) Fraction of PH3 positive cells upon respective treatment. N = 23 and 15 embryos respectively for uninjected and LIC2 MO treatment from 2 experiments. (**E**) Confocal images of surface blastomeres showing longer mitotic spindles (white dotted line), control and unfocused spindle poles (white arrows) and fragmented spindle poles (multiple white arrows) in zLIC1 and zLIC2 morphants. Chromosomes (DAPI, blue), spindle poles (γ-tubulin, green) and microtubules (α-tubulin, red) are immunostained as indicated. (**F**) Average spindle length in surface blastomeres. n = 139 control cells (30 embryos), 118 metaphase cells for LIC2 depletion (26 embryos) and 52 metaphase cells (10 embryos) for LIC1 depletion, across a minimum 3 experiments each. (**G**) Fraction of blastomeres showing chromosome congression defects from the embryos in F. (**H**) Fraction of blastomeres showing spindle pole focusing defects upon LIC2 depletion. n = 69 control metaphase cells (12 embryos) and 95 metaphase cells (16 embryos) across a minimum of 3 experiments each. (**I**) Fraction of blastomeres showing spindle pole fragmentation upon LIC1 depletion. n = 52 metaphase cells (10 embryos) across 3 experiments. Error bars are mean +/− SD.

We probed the mechanistic basis for the developmental delay upon LIC2 depletion, surmising that like in mammalian cells, LIC2 plays important roles in mitotic progression in zebrafish embryos. We immunostained embryos for microtubules, spindle poles and chromosomes and performed confocal microscopy followed by 3-dimensional image reconstruction to visualize the surface of whole embryos. LIC2 depleted embryos were of approximately similar size as control embryos, but were distinctly oblong and deformed (top view), in contrast with control embryos that were spherical (Fig. 7A). Individual blastomeres of LIC2-depleted embryos were larger and misshapen as compared to control embryos (Fig. 7A). LIC2 depleted furrowed embryos showed a 5–6-fold reduction in the total number of nuclei compared to control embryos at the same time post injection (Fig. 7B), suggesting a mitotic delay. The mitotic delay was corroborated by a visibly higher fraction of phosphohistone 3 (PH3) positive surface blastomeres in LIC2 depleted furrowed embryos (Fig. 7C,D). Microscopic analysis revealed that metaphase cells from LIC1 and LIC2 depleted embryos showed significantly elongated spindles compared to control embryos (Fig. 7E,F), similar to the elongated spindles seen in mammalian cells (Supplementary Fig. S3). LIC2 depletion, but not LIC1 depletion also led to uncongressed chromosomes in a significant fraction of blastomeres of furrowed embryos (Fig. 7E,G) as seen in mammalian cells (Supplementary Fig. S3). Additionally, LIC2 depleted embryos showed bipolar spindles with unfocused spindle poles (Fig. 7E,H) similar to the pole dispersion phenotype seen in mammalian cells (Supplementary Fig. S2) while LIC1-depleted embryos showed fragmented spindle poles (Fig. 7E,I). These results are consistent with the recently demonstrated roles for LICs in maintaining spindle pole integrity^12^. Although the gross morphology of the LIC1/2 depleted morphants is similar (Fig. 6C), the underlying mitotic phenotypes appear to be distinct. Together, our results demonstrate that LIC2 plays conserved roles in spindle organization and mitotic fidelity during embryogenesis, and is essential for governing early cell divisions and embryonic development in vertebrate embryos.

## Discussion

**Figure 8:**
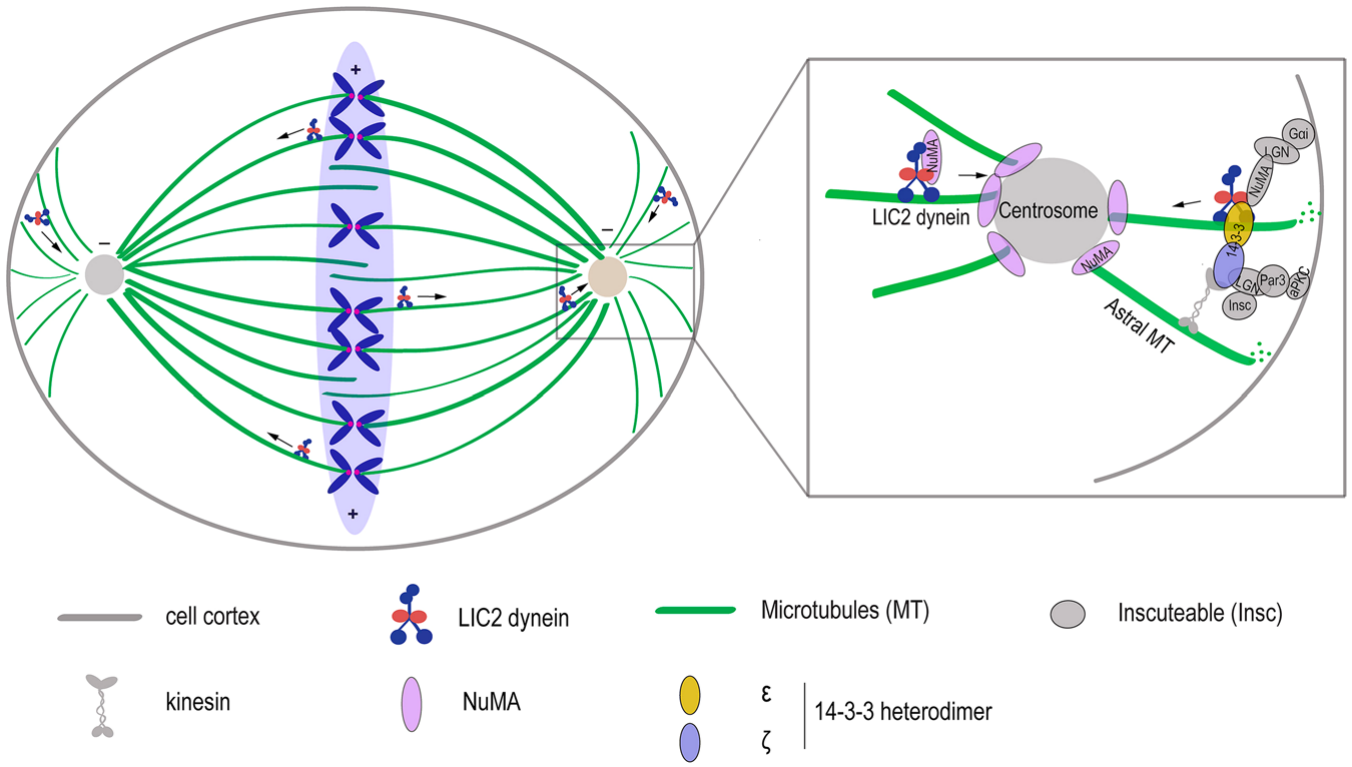
A model for LIC2-dynein based control of mitotic spindle orientation. LIC2-dynein interacts with NuMA, Par3 and 14-3-3 and anchors astral microtubules to the cortex. LIC2-dynein also transports cortical NuMA along astral microtubules to the spindle poles, thus helping to focus microtubule minus ends at the centrosome. LIC2 dynein may stabilize the bridging of the NuMA- and Par3-mediated spindle orientation pathways through its interaction with the 14-3-3 ε/ζ heterodimer.

The extensive mitotic localization of LIC2 prompted us to explore its functional repertoire during vertebrate mitosis. Our results reveal pleiotropic functions of LIC2-dynein during mitosis and uncover protein interaction specificity among the LICs that could explain these functions (Fig. 8). LIC2-dynein is required for spindle orientation via its ability to anchor astral microtubules at the cortex through its interaction with cortical NuMA, Par3 and 14-3-3 proteins. LIC2 also transports NuMA to spindle poles, which in turn is responsible for maintaining proper centrosomal focusing. LIC1 plays a stronger, perhaps mechanistically distinct role in this function. LIC2 is responsible for congression of condensed chromosomes to the equatorial plate and regulates spindle length by shortening it. It is also noteworthy that we quantified the mitotic phenotypes only in cells that had spent abnormally prolonged periods in mitosis upon LIC1/2 depletion. The mitotic phenotypes of spindle mis-orientation and pole fragmentation were however a direct consequence of LIC2/LIC1 depletion and not of the prolonged arrest itself (Supplementary Fig. S4).

A major advance from this study about our mechanistic understanding of mitotic dynein is the discovery of a dominant role for LIC2-dynein in regulating spindle orientation (Fig. 8). The orientation of cell division plays key roles in regulating stem cell self-renewal vs. differentiation^57–60^, and spindle mis-orientation is suggested as a cause for disorders like polycystic kidney disease^61–66^. The spindle mis-orientation observed upon LIC2 depletion is stark (Fig. 3), with a 4-fold increase in the fraction of cells showing appreciable spindle tilt (>20 degrees). In contrast, LIC1 depletion shows a mild defect, highlighting the dominance of LIC2 in influencing spindle orientation. The orientation defects were not due to any defects in adhesion to the substratum (Supplementary Fig. S3). Both LIC2 and the mitotic regulator NuMA start appearing prominently at spindle poles simultaneously in prometaphase^31, 32^, making LIC2 a prime suspect in linking NuMA to cytoplasmic dynein. A major dynein-dependent mechanism regulating spindle orientation is likely to be through the interaction of LIC2-dynein with NuMA at the pole-proximal cortex (Fig. 8), although a dynein-independent role for NuMA in orienting the spindle has also been recently demonstrated^67^. NuMA is a critical component of the cortically localized LGN-Gαi-NuMA complex that attaches to astral microtubules and mechanically anchors the centrosome to the cortex^14, 43^, thus stabilizing spindle orientation. LIC2 depletion also led to elevated levels of cortical NuMA and a concomitant reduction in polar NuMA levels at the upper cortex and upper pole respectively of mis-oriented spindles (Fig. 4 and Supplementary Fig. S5). The distribution of cytoplasmic dynein at the cortex in mitotic cells is naturally asymmetric, with more dynein concentrated at the cortices corresponding to the two poles^36^. Therefore, dynein-based transport of cortical NuMA towards the pole^43^ would also be asymmetrically affected upon LIC2 depletion. This could explain the differential NuMA levels upon LIC2 depletion at the upper and lower poles. The altered NuMA levels at these sites upon LIC2 depletion are attributable to the impaired transport of NuMA from the cortex to the pole due to the inability of dynein to capture cortical NuMA. In support of this hypothesis, the levels of cortical dynein reduced appreciably upon LIC2 depletion, (Fig. 5), demonstrating that LIC2-dynein is anchored at the cortex at least partially through its interaction with NuMA. This result also indicated that a larger fraction of cortical dynein consists of the LIC2-dynein subpopulation. Development of more robust methods for quantification of cortical fluorescence would help reveal the levels of the two dynein subpopulations with greater precision. The majority of pole-localized NuMA is recruited by diffusion from the cytoplasm in a dynein-independent manner, while a smaller but significant fraction is deposited at poles by dynein mediated transport^37, 45, 46, 48^. Our results indicate that LIC2-dynein could account for the majority of dynein-mediated poleward transport of NuMA, which originates primarily from the cortex.

The other major cortically localized protein complex that ensures spindle orientation is the Par3-aPKC-LGN complex^28, 29^. This pathway has been shown to be partially sufficient for orienting the spindle, by interacting with Dlg and kinesin to capture astral microtubules near the cortex^28^. Interestingly, Par3 has been demonstrated to exclusively interact with LIC2 but not with LIC1 in interphase to position the centrosome^51^. We found that the Par3-LIC2 interaction is also preserved in mitosis (Fig. 5D). The 14-3-3 heterodimer consisting of its ε and ζ proteins is known to interact with dynein and kinesin, thus bridging the NuMa-LGN and Par3-aPKC pathways, two important spindle orientation pathways that together achieve complete orientation^28^. Indeed, the 14-3-3 heterodimer interacted very robustly and specifically with LIC2 but not with LIC1 (Fig. 5D), demonstrating that LIC2-dynein could assist the 14-3-3 proteins in bridging these two pathways. The strong interaction of the 14-3-3 proteins with LIC2 suggests that they may interact with LIC2-dynein upstream of the other effectors of the two orientation pathways, although this remains to be demonstrated. The specific binding of LIC2-dynein to Par3 and especially to the 14-3-3 heterodimer illuminates the two major molecular networks that LIC2-dynein associates with to mediate spindle orientation.

LIC2 depletion also led to elongated spindles, with the poles juxtaposed with the cortical membrane (Supplementary Fig. S9). In this scenario, the centrosomal NuMA could anchor the poles to the cortex directly through its interaction with membrane phospholipids^68^. This attachment may happen at a later stage at random sites on the cortex, leading to mis-oriented spindles. LIC2 depletion also led to chromosome mis-congression in approximately 50% mitotic cells (Supplementary Fig. S9). The mitotic arrest seen in these cells is likely to be due to an active spindle assembly checkpoint signal, as expected due to their inability to establish proper inter-kinetochore tension^53, 69, 70^.

LIC2 was crucial for regulating the early divisions in zebrafish embryos. Our zebrafish results mirror the LIC1- and LIC2- specific phenotypes observed in mammalian cells. We found that both LICs were expressed from the earliest stages of the embryo (Fig. 6) even before zygotic gene expression commences^71, 72^, indicating maternal inheritance. The furrow phenotype seen at the mid-blastula transition (MBT) stage (Fig. 6) upon LIC2 depletion could have many implications. The altered cellular and embryonic shape (Figs 6 and 7) could cause defects in subsequent morphogenetic processes like gastrulation, and alter the cell fate imparted on these blastomeres, possibly resulting in defects later during development. Severe developmental delays were indeed observed at the blastula stage upon zLIC2 depletion, with stunted head and tail bud regions seen at 1 dpf (Fig. 6). An important underlying basis for the developmental delay is defective cell proliferation (Fig. 7). Upon LIC2 depletion, we readily observed phenocopy of various mitotic spindle defects seen in mammalian cells during the blastula stage of control embryos. Mitotic spindles were significantly elongated (Fig. 7), which could result in cell shape changes of individual blastomeres and by extension, in the oblong morphology of the whole embryo, as opposed to a more spherical shape of control embryos (Fig. 6). The multiple mitotic defects seen in zebrafish embryos are likely to cumulatively cause the developmental delays observed upon LIC2 depletion at 1 dpf (Fig. 6) and suggested a strong conservation of the mitotic functions of LIC2-dynein across vertebrates. The long-term developmental consequences of LIC2 depletion and the molecular mechanisms that enable crosstalk with extracellular developmental cues remain to be elucidated.

LIC1 and LIC2 are believed to occupy cytoplasmic dynein in mutually exclusive complexes^25^, conceivably to perform at least a set of distinct functions. In support of this hypothesis, it was recently reported that LIC1-and LIC2-dynein show differential effects on inactivating the spindle assembly checkpoint^32^. Here we demonstrate that a prominent share of mitotic dynein functions is performed by LIC2-dynein, which plays dominant roles in regulating chromosome congression and spindle length and ensures proper spindle orientation. LIC1-dynein plays minor roles in all of these functions, perhaps through other mechanisms. We show that only LIC2-dynein governs spindle orientation through its exclusive interactions with 14-3-3, Par3 and NuMA. The binding of mitotic LICs to exclusive cargoes has some precedence; LIC1 exclusively binds to the key mitotic regulator pericentrin^25, 73, 74^. It is instructive that invertebrates have only one LIC gene, suggesting that vertebrate LIC1 and LIC2 have evolved in a divergent manner to perform more complex functions. While both LICs have been shown to be important for maintaining spindle pole integrity in vertebrates^12^, it is likely that they operate at least partially through distinct biochemical pathways. Given the exclusivity of the LIC1-pericentrin interaction, it is conceivable that LIC1-dynein contributes to spindle pole integrity by ensuring proper formation of the pericentriolar material, in addition to its role in centriole cohesion^12^. LIC1 also appears to be more relevant in some interphase functions, where it localizes to centrosomes and influences Golgi complex organization, a function in which LIC2 plays no significant role^4, 30, 33^. It is logical to surmise that the distinct mitotic functions of the two LICs are governed by unique biochemical interactions with other proteins, as shown here for LIC2-dynein in spindle orientation. Elucidation of these molecular mechanisms would illuminate mechanistic details governing the biology of the two LIC homologues.

## Methods

### Cell culture and cell synchronization

HeLa cells (Sigma Aldrich/ECACC), U2OS cells and hTertRPE1 cells (gifts from Stephen Doxsey) were grown in appropriate culture media. The mfGFP-IC74 Hela stable cell line (gift from Takashi Murayama) was grown in medium supplemented with hygromycin B. The H2B-mCherry-GFP-α tubulin line (gift from Daniel Gerlich) was grown in medium supplemented with hygromycin B and puromycin. Nocodazole (Sigma) was used at 150 nM (HeLa) or 400 nM (U2OS) for 12 hours to arrest cells in prometaphase. For obtaining metaphase cells, the prometaphase arrest was released by washing the cells extensively with PBS (phosphate buffer saline) and harvesting after 1 hour, or by treatment of nocodazole-released cells with MG132 (10 µM). Dynein inhibitor ciliobrevin D (Millipore) dissolved in DMSO was used as a positive control and added to 60–70% confluent cells at 30 μM and 50 μM concentration in fresh media followed by time-lapse imaging.

### Plasmid constructs, siRNAs and transfection

Full length rat LIC2 cDNA was cloned into the pCMV 3Tag 3B vector (Agilent Technologies) using EcoR1 and Xho1 restriction sites and sequenced. Plasmid transfection was performed using Lipofectamine 2000 (Invitrogen) as described in the manual. SiRNAs against different human genes were synthesized by Dharmacon. The detailed sequences and working concentrations of individual siRNAs were as follows–LIC1: GAAAGUUUGUACAUGAGAA (100 nM), LIC2a: ACCUCGACUUGUUGUAUAA (100 nM), LIC2b: GCCGGAAGAUGCAUAUGAA (100 nM), GFP (negative control): CAUGAAGCAGCACGACUUC (100 nM). All the siRNA sequences were previously used and published by different groups^30, 33^. siRNA transfection was achieved using Dharmafect 1 (Dharmacon/Thermo Scientific) for 48 hrs. Negative controls for siRNA treatment used either GFP siRNAs or mock transfections. During co-depletion of LIC1 + 2, 100 nM each of the two siRNAs were used. For rescue experiments in cell lines, plasmids were transfected on day 1 followed by siRNA transfection on day 2 and observation at 48 hrs after siRNA transfection. Metaphase index was calculated by counting metaphase cells as a fraction of total cells under a fluorescence microscope or confocal microscope to visualize chromosomes, which were stained by 4′, 6-diamidino-2-phenylindole (DAPI) or Syto 13 (Invitrogen) respectively.

### Antibodies

Primary antibodies against the following antigens were used: LIC1 (Thermo Scientific PA5-31644); LIC2 (Abcam ab174895, ab178702), γ-tubulin (A302-631A, Bethyl laboratories); NuMA (Novus Biologicals NB500-174); α-tubulin (DM1α, T9026), β–actin (A3835), Flag M2 (F1804) monoclonal antibodies from Sigma; IC-74 monoclonal antibody (Abcam ab23905), Par3 (Milllipore, 07-330), 14-3-3 ζ (Abcam, ab124431), 14-3-3 ε (Abcam, ab43057). HRP conjugated anti-mouse (715-035-150) and anti-rabbit (711-035-152) secondary antibodies for Western blotting and fluorophore attached DyLight 488 (115-485-003), Cy3 (111-165-144), Cy5 (109-175-008) secondary antibodies for immunofluorescence analyses were purchased from Jackson Immunoresearch.

### Western blotting

The following antibody dilutions were used for Western blotting: LIC1- 1:1000, LIC2–1:1000, β-actin- 1:2000, IC74–1:1000, 14-3-3 ε–1:1000, 14-3-3 ζ–1:200; Par3 1:400, Flag M2 -1:10000, anti-mouse HRP 1:10000, anti-rabbit HRP- 1:10000. Cell lysates were prepared by directly adding Laemmli buffer into the culture plate and boiling the sample at 95 °C for 5 min. Samples were run on SDS-PAGE, followed by transfer of proteins on to polyvinylidene difluoride (PVDF) membrane (Millipore). Blots were blocked with 5% skimmed milk followed by incubation in primary antibody for 1 hr at room temperature or at 4 **°**C overnight, washed and incubated with secondary HRP conjugated antibodies for 1 hr at room temperature, and washed extensively. The chemiluminescene signal was developed using the Luminata Forte reagent (Millipore) and captured in the Image Quant 4000 (GE). Densitometric quantification of band intensities was performed using the GE Image Quant 4000 platform.

For zebrafish samples, embryos were dechorionated manually and washed in fresh E3 media. Embryos older than 1dpf were also de-yolked manually and washed in E3 media. The embryos were lysed in Laemmli buffer and boiled at 95 °C for 5 min. Protein estimation was performed using the CBX protein estimation assay (G-Biosciences). The proteins were resolved by SDS-PAGE, transferred to PVDF membranes and probed with the following antibodies at 4 °C overnight: LIC2 (1:500, Abcam), β-actin (1:1000, Sigma), anti rabbit (1:10,000, Jackson Laboratories) and anti-mouse (1:10,000, Jackson Laboratories) HRP conjugated secondary antibodies were used along with Precision Protein strepTactin HRP conjugate (Biorad). The blots were developed and visualized as mentioned above.

### Immunoprecipitation and affinity purification

The IC-74 antibody was used for immunoprecipitation experiments. A Streptactin-HP (GE) affinity column was used for purification of the MTAP-mVenus-hLIC1 and –hLIC2 protein from a stably expressing cell line, using the Streptavidin-binding-protein tag imparted by the MTAP-mVenus vector^75^. Mitotically enriched cells were suspended in lysis buffer containing 50 mM Tris pH 7.5, 125 mM sodium chloride, 1mM EGTA, 0.2 NP-40, 5% glycerol, protease inhibitors and phosphatase inhibitors (Roche/Pierce) and incubated for at least 20 minutes on ice. Whole cell lysates were pre-cleared by incubating with protein G sepharose beads (GE). Protein G sepharose beads (GE) were used for immunoprecipitation. Primary antibodies were crosslinked to protein G sepharose beads by incubating antibody with beads for 1 hour at 4 °C followed by washing to remove unbound antibody. Pre-cleared cell lysates were subjected to immunoprecipitation by incubating suitable beads with pre-cleared lysates for 6 to 8 hours at 4 °C with tumbling. Immunoprecipitated samples were resolved by SDS PAGE followed by Western blotting.

### Immunofluorescence staining

The following antibody dilutions were used: LIC1–1:250, LIC2–1:100, IC74–1:500, NuMA–1:100, α-tubulin–1:1000, γ-tubulin–1:500. HeLa cells were grown on glass coverslips, washed with PBS, and fixed in 4% formaldehyde or chilled methanol. Fixed coverslips were incubated with blocking buffer (PBS + 1% bovine serum albumin + 0.5% Triton X-100) for 1 hour, followed by incubation for 1 hour with primary antibody, washed and incubated with secondary antibodies in a humidified chamber. DAPI staining was performed (1:10,000 concentration of a 5 mg/ml stock solution) for 1 minute, cells washed in PBS and water, and mounted on a glass slide using mounting medium (Prolong Gold, Invitrogen). Imaging of fixed coverslips was performed after drying the mounting medium.

For zebrafish embryos: whole embryos were fixed in 4% formaldehyde in PBS at 4 °C overnight followed by post-fixing in 100% methanol until use. Fixed embryos were rehydrated and washed in PBS, dechorinated and blocked with 1% PBSAT (1% bovine serum albumin in 1X PBS + 0.1% Triton X-100) for 2–4 hours at room temperature. The following primary antibodies were used: gamma tubulin (1:1000, Bethyl labs), α tubulin (1:250, DSHB) and phosphohistone 3 (1:250, Thermo Scientific). Primary and secondary antibody incubations were carried out in 1% PBSAT at 4 °C overnight with 1X PBSAT washes between incubations. The embryos were washed in PBS, stained with DAPI, washed again in PBS and mounted for microscopic examination.

### Microscopy

#### Immunofluorescence imaging of cells

Fixed cell image acquisition was performed on a Leica TCS SP5 II (with Leica DFC 360FX camera, version: FCAM 2 V1.0 in fluorescence mode or using PMTs in laser illumination mode) or Leica TCS SP8 laser scanning optical confocal microscope using a HCX PL APO CS 63X-1.4 numerical aperture oil immersion objective. All acquisition settings were identical for control and test samples. Only metaphase cells (judged by DAPI staining) were imaged to analyze spindle orientation, chromosome congression and spindle length. Immunostained embryos were visualized under a 40X Plan-Apochromat objective of Leica TCS SP5 II confocal microscope. The number of cells and embryos counted per experiment for statistical analysis is indicated in the figure legends. Error bars depict standard deviation across a minimum of 3 experiments unless otherwise specified.

#### Time lapse imaging

HeLa cells were grown on coverslips and suitably treated. The coverslips were placed on custom-designed aluminium slide containing chambers. Time-lapse imaging was performed for 12 hours with a 2.5 or 5-minute time interval between successive frames at four different positions on each coverslip in bright field or laser fluorescence modes, using a 20X, 40X or 63X Plan-Apochromat objectives. In some experiments, Labtek chambered cover glass (Nunc) were used.

#### Gridded coverslip experiments

HeLa cells were grown on gridded coverslips (Electron Microscopy Sciences). After 36 hrs of siRNA transfection the coverslips were followed by time-lapse imaging as described above. The same gridded coverslips were fixed and used for immunostaining and imaged as described above. The grids on the coverslips were used to locate the very same cells that got arrested in mitosis for prolonged periods (over 1.5 hours); only these cells were used for further fixed cell imaging following immunostaining.

#### Image analysis

Fluorescence image analysis was performed on the Imaris software suite (version 5.7, Bitplane), Leica offline image analysis software (LAS) and ImageJ. For spindle orientation measurements, immunofluorescence images were reconstructed in 3 dimensions using Imaris. The angle made by the spindle axis with the substratum was measured using ImageJ software by the method described earlier^63, 76, 77^. Further, to show orientation defects in live cells, we observed the uneven flattening pattern of mitotic cells as they exit mitosis^63^ by visual observation of time-lapse movies of dividing cells. Dividing Hela cells round up during mitosis and flatten onto the substratum almost simultaneously as they exit mitosis (movie 11). The two daughter cells generated from mis-oriented poles (such as upon LIC2 depletion) do not re-flatten at the same time (movie 12); the daughter cell with the pole nearer to the substratum flattens first followed by the other, which stays rounded for longer and flattens later. NuMA intensity at the cortex was measured by line scans and ROI intensity measurements^36^ on the LAS Leica offline analysis software (LAS). For measuring NuMA levels at the poles, images were reconstructed in Imaris, a sphere of diameter 4.5 µm was drawn around the spindle poles and fluorescence intensity within the sphere measured. Intensities of mf-GFP-IC74 at the cortex were quantified as previously described^36^ using four rectangular boxes drawn at the cell cortex. Spindle lengths were measured as the distance between the centers of two spindle poles (visualized using γ-tubulin staining) in either the LAS or Imaris software suites. For zebrafish embryo imaging, the z-stack images acquired were processed on the LAS Leica offline software. Imaris 3D imaging software suite was used to generate 3D reconstructions of the entire surface of embryos. The 3D reconstructions were used to count the total number of cells in the embryos, calculate spindle length, chromosome congression and spindle pole-focusing defects.

### Zebrafish lines, morpholino injection and characterization of phenotype

Tuebingen strain zebrafish were raised according to standard protocols as described earlier^78^. Embryos were obtained from natural spawning of adult fish. The embryos were kept at 28.5 °C and staged according to hours post fertilization^79^. Morpholinos used (all from Gene Tools LLC) were as follows: TTCTTCTCTAAAACGGGAGCCATCT (zLIC2 translation blocker), GTTGAAGTTTTTGAGTTACCTTTGT (zLIC2 splice blocker), TTgTTgTCTAtAACGGcAcCCATCT (zLIC2 mismatch) and GTGTATTTCTGCCCGTCGTCGCCAT (zLIC1 translation blocker). p53 morpholino and standard control morpholino were used as negative controls. One-cell stage embryos were injected with LIC2 translational blocker (5 ng/15 ng) and 15 ng of other morpholinos. Gross anatomical defects were visualized around 3.3 hpf and at 1 dpf with a Zeiss SMZ Stemi 2000C microscope. Morphant embryos collected around 3.3 hpf (corresponding to blastula stage of control embryos) were analyzed by high resolution imaging as described above. LIC depletion was assessed by immunoblotting (LIC2 translation blocker) or reverse transcriptase PCR (LIC2 splice blocker). The following primers used for PCR: CGCTGTAGTGTCTGGATCCTGG (forward primer) and GCCTTAGTGGTGGAGAAGGACG (reverse primer). For rescue experiments, rLIC2 mRNA was synthesized using the mMESSAGE mMACHINE kit (AM1348, Ambion) and 25 pg of mRNA injected per embryo. For real time PCR, total RNA was isolated from different developmental stages of wild type Tuebingen zebrafish using a RNA isolation kit (Promega) and cDNA synthesized (using BioRad iScript cDNA synthesis kit). The following primers were used for the real time PCR for zLIC1 (Fwd GAAGAGTACATGAAGGGACGAG, Rev CACTACAAACAGAATCAGCGAG) and housekeeping gene control ribosomal protein L (zRPL13a): Fwd TCTGGAGGACTGTAAGAGGTATGC and Rev AGACGCACAATCTTGAGAGCAG. LIC1 real time expression data is normalized to the expression at the 1-cell stage.

### Statistical analysis

The number of cells counted per experiment for statistical analysis is mentioned in the respective figure legends. The graphs in all figures are depicted with error bars, mean of at least 3 experiments +/−SD or SEM, unless stated otherwise. Statistical significance was calculated by Student’s t-test or one-way ANOVA with Tukeys comparison method or Kruskal Wallis test. Scale bars (μm) for images are indicated in the respective legends. Graphs were created using the GraphPad PRISM software.

### Institutional approvals

All experiments and protocols other than those involving live zebrafish were performed with the approval of the institutional biosafety committee of the Regional Centre for Biotechnology, India. All live zebrafish experiments were performed according to protocols approved by the Institutional Animal Ethics Committee (IAEC) of the CSIR-Institute of Genomics and Integrative Biology, India.

## Electronic supplementary material

Supplementary Figures and Legends

Supplementary Movie 1

Supplementary Movie 2

Supplementary Movie 3

Supplementary Movie 4

Supplementary Movie 5

Supplementary Movie 6

Supplementary Movie 7

Supplementary Movie 8

Supplementary Movie 9

Supplementary Movie 10

Supplementary Movie 11

Supplementary Movie 12

Supplementary Movie 13

Supplementary Movie 14

Supplementary Movie 15

Supplementary Movie 16

Supplementary Movie 17

Supplementary Movie 18

Supplementary Movie 19

Supplementary Movie 20

Supplementary Movie 21
