# Supplementary material for: The Light Intermediate Chain 2 Subpopulation of Dynein Regulates Mitotic Spindle Orientation
Source: Sci Rep. 2016 Dec 23;6:22. doi: 10.1038/s41598-016-0030-3 (PMC5431351; doi:10.1038/s41598-016-0030-3)
Supplement: Supplementary file 1 — Supplementary Figures and Legends [file 41598_2016_30_MOESM1_ESM.pdf]

Supplementary information

The Light Intermediate Chain 2 Subpopulation of Dynein Regulates Mitotic Spindle Orientation

Running title: Dynein LICs distribute mitotic functions.

Sagar Mahale[a, d, *], Megha Kumar[a, *], Amit Sharma[a, d, *], Aswini Babu[b, c], Shashi Ranjan[b], Chetana Sachidanandan[b, c] and Sivaram V S Mylavarapu[a, d, §].

Supplementary figures and legends

Supplementary figure S1

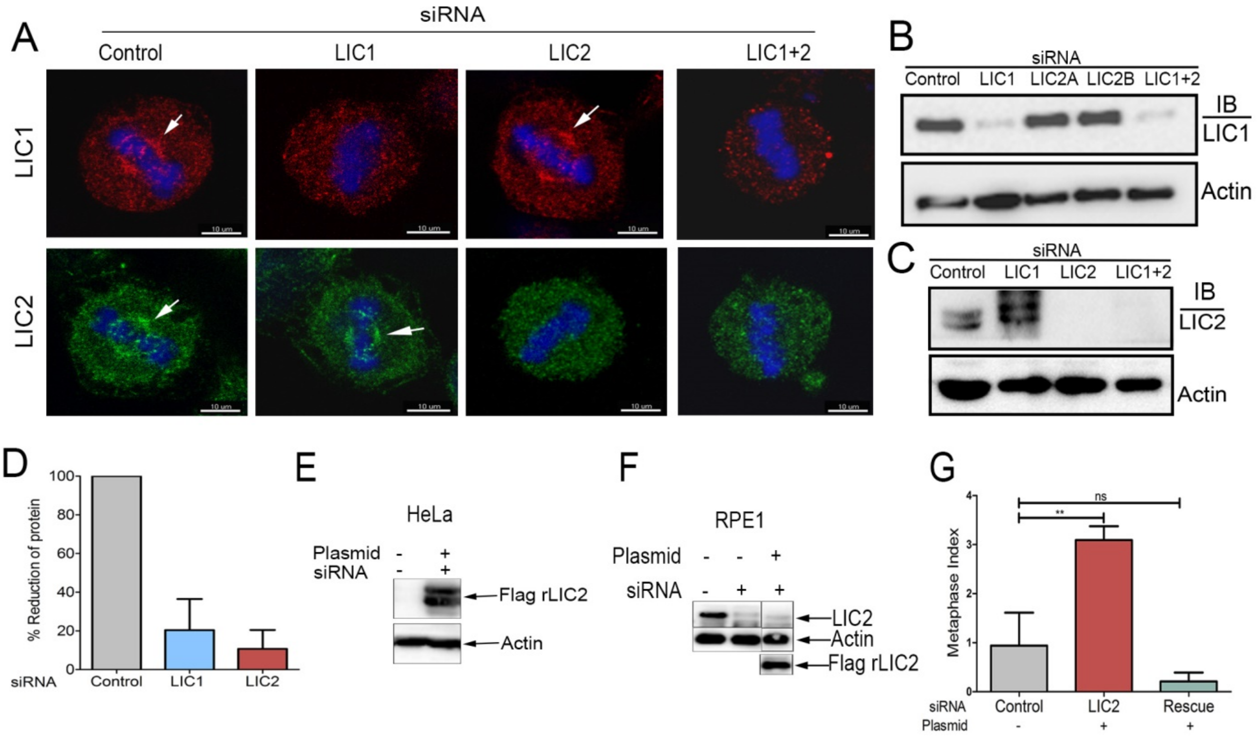

Supplementary figure S1*: *Characterization and specificity of antibodies and protein depletions

A) Confocal immunofluorescence images of HeLa cells depleted of LIC1/ LIC2 using sequence specific siRNA and stained with respective antibodies. B) & C) Western blots showing siRNA mediated LIC1 and LIC2 specific knockdown. Actin is the loading control. D) Graph showing efficiency of protein depletion upon respective siRNA treatments from densitometric quantification of immunoblot band intensities. n = 3 experiments. E). Western blot showing the transgenic expression of FLAG tagged-rLIC2 in hLIC2 depleted HeLa cells. (lane 1: control, lane 2: FLAG-rLIC2 transfected). F) Depletion of endogenous hLIC2 and transgenic expression of FLAG-rLIC2 in RPE1 cell line (lane 1: control, lane 2: LIC2 depleted, lane3: depletion of endogenous LIC2 and transgenic expression of FLAG-rLIC2. G) Mitotic index in RPE1 cells treated with hLIC2 siRNA and/or transgenic FLAG-rLIC2 (n= 3 experiments, approximately 500 cells were counted for each experiment).

Supplementary figure S2

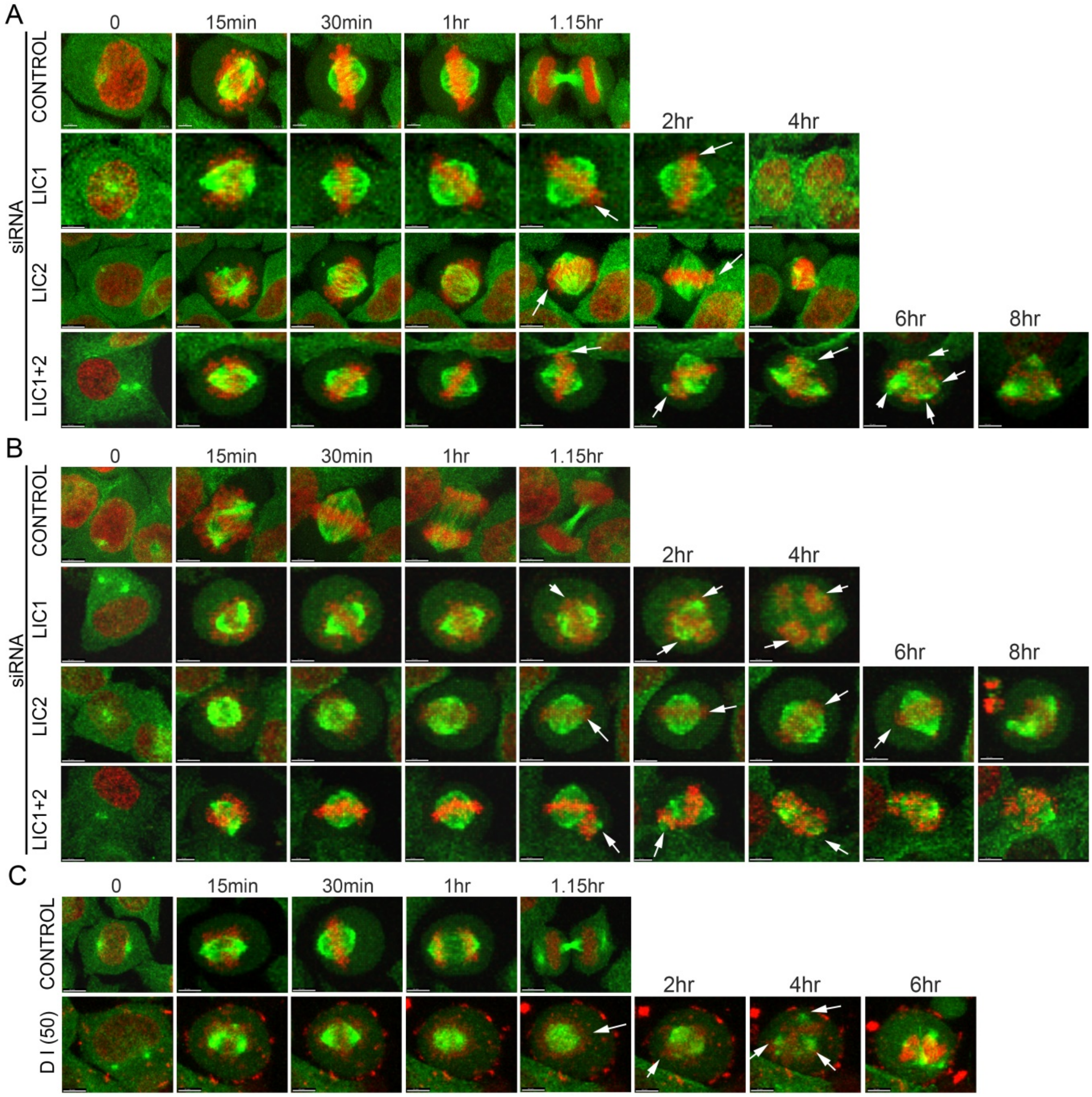

Supplementary figure S2*: *Additional figure with representative images in support of figure 1 showing that LICs are required for mitotic progression in HeLa cells.

A & B) Still images from movies featuring different siRNA treated HeLa cells expressing H2B-mCherry and GFP-α-tubulin (control, LIC1, LIC2 and LIC 1 + 2 depleted) showing prolonged arrest in mitosis. Time points indicate time after nuclear envelope breakdown. C) Still images from movies representing the effect of dynein inhibitor (DI) at 50 μM concentration with DMSO as control.

Supplementary figure S3

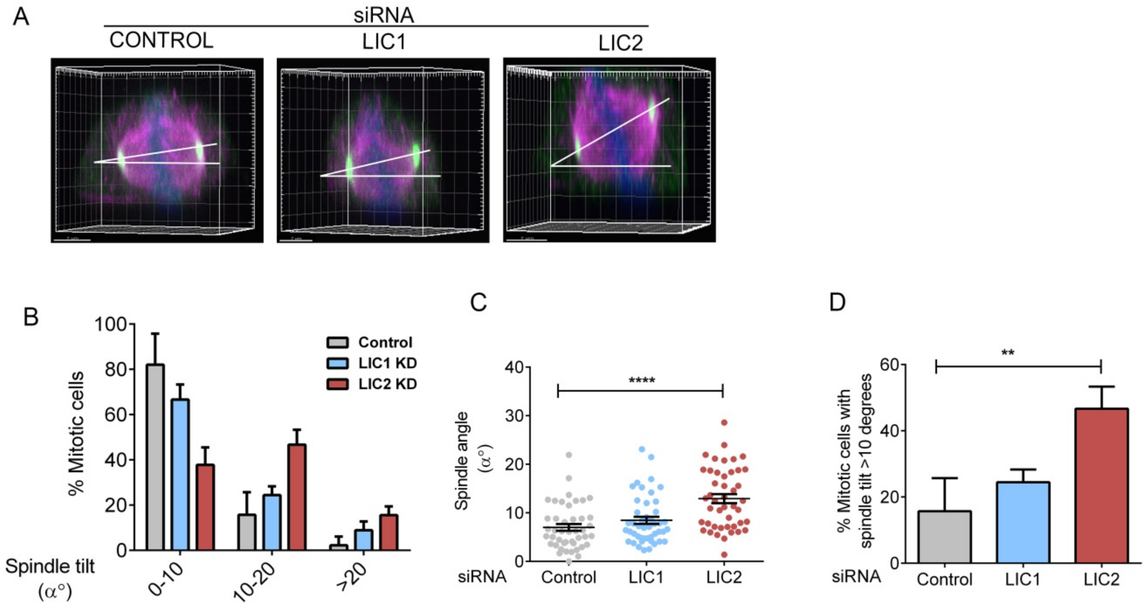

Supplementary figure S3: LIC2 is required for spindle orientation independent of adhesion to the substratum

All cells were grown on collagen-coated cover slips to ensure proper adhesion to the substratum, n = 3 experiments, 10 metaphase cells per experiment. A) Spindle angle in representative mitotic HeLa cells, depicted from 3D reconstructions of confocal z-stacks, after respective siRNA treatment. B) Distribution of spindle angle across mitotic cells upon respective siRNA treatment. C) Spindle angle (tilt) upon respective siRNA treatment. D) Fraction of metaphase cells with a spindle angle of > 10 degrees. Error bars = +/- SEM from 3 independent experiments.

Supplementary figure S4

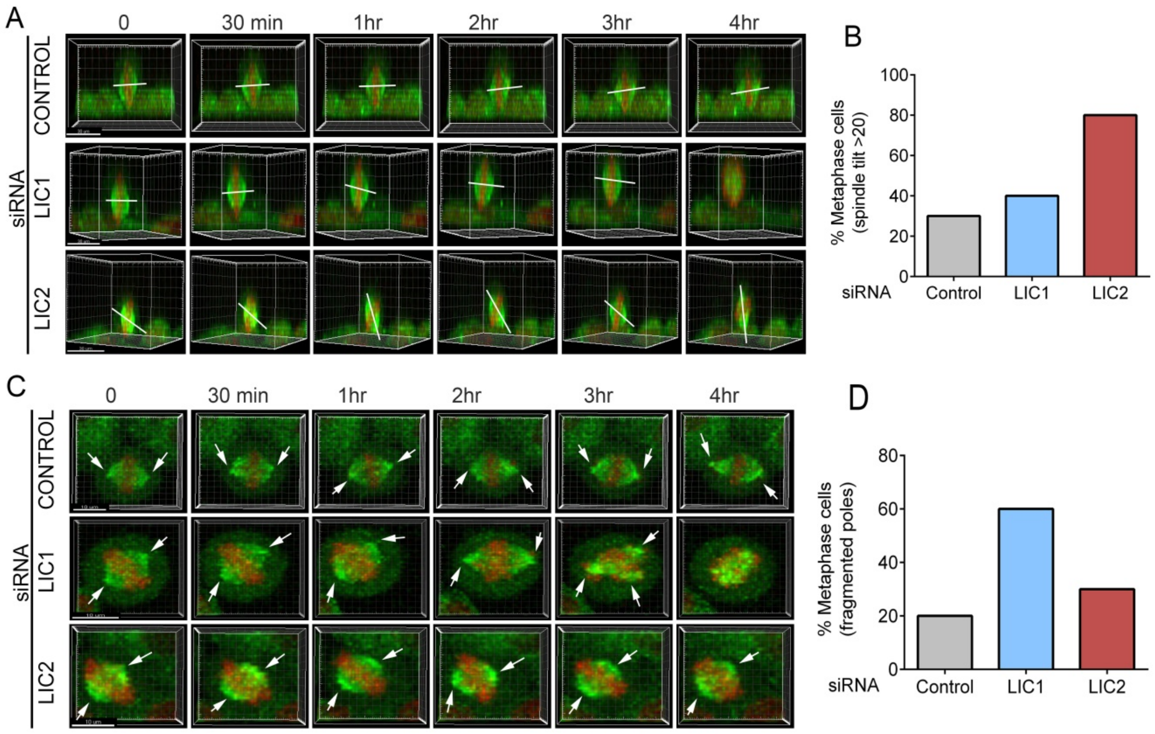

Supplementary figure S4: Prolonged mitotic arrest does not lead to spindle mis-orientation or spindle pole fragmentation

HeLa cells expressing GFP-tubulin (green, microtubules) and mCherry Histone 2B (red, chromosomes) were treated with respective siRNAs for 48 hours, arrested in metaphase using treatment with MG132 (10 µM) and imaged live in the confocal microscope for upto 4 hours. N = 2 independent experiments, 10 metaphase cells per experiment. A) Stills from representative live movies after respective siRNA treatment showing the spindle angle with respect to the substratum (white line). B) Fraction of metaphase cells with spindle angle > 20 degrees. C) Stills from representative live movies after respective siRNA treatment showing spindle pole fragmentation. Arrows indicate spindle poles. D) Fraction of metaphase cells with fragmented spindle poles.

Supplementary figure S5

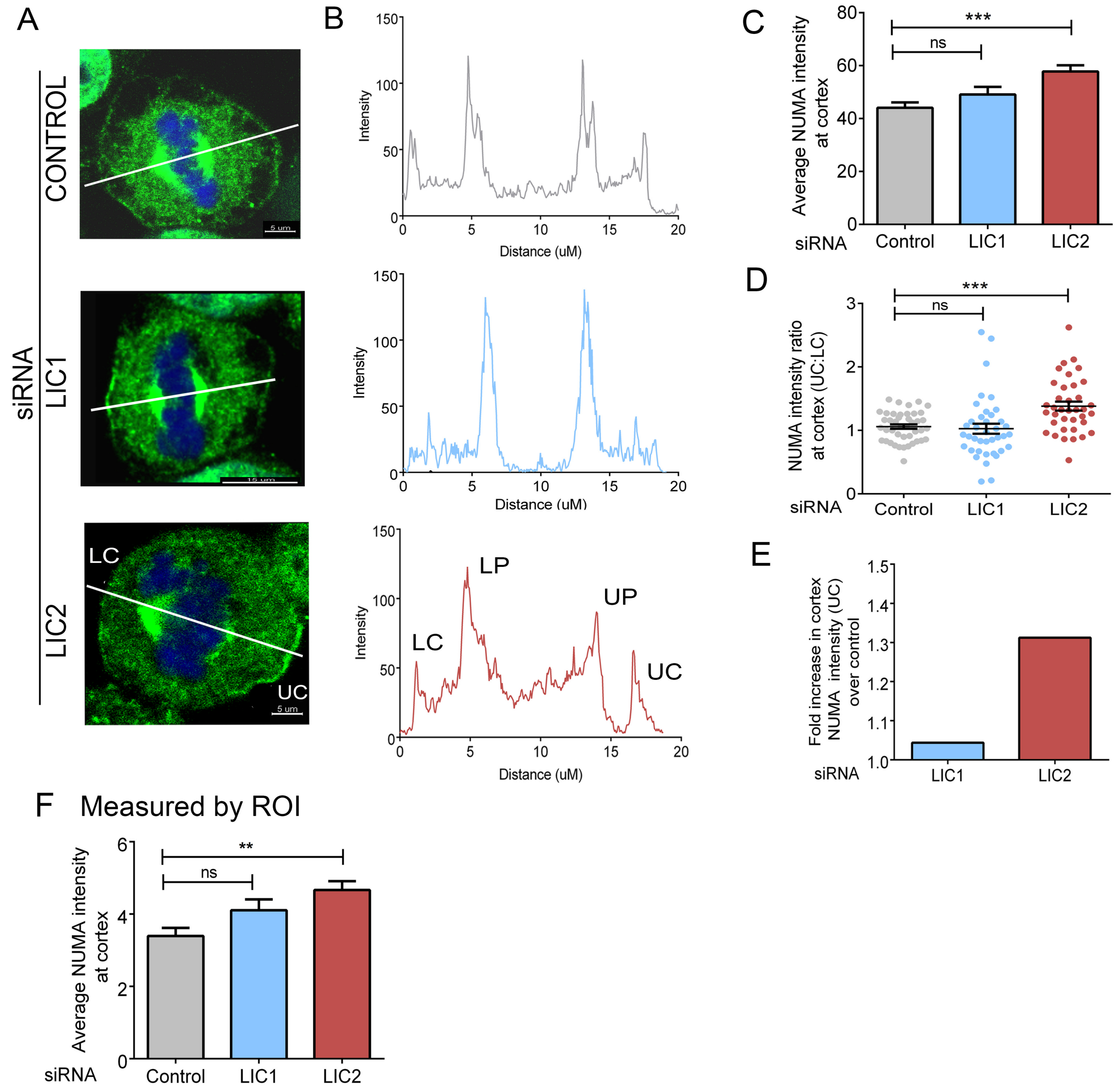

Supplementary figure S5: LIC2 transports NuMA from the cell cortex.

A) Representative images showing line scan measurements of NuMA intensity along the spindle axis. B) Graphical representation of the line scans from A. C) Average NuMA intensity at the cortex from quantification using line scans. D) Ratio of fluorescence intensities (UC/ LC). Error bars are mean +/- SEM from 3 independent experiments, at least 20 metaphase arrested cells per experiment for C and D. E) Fold increase in average NuMA levels at the upper cortex upon LIC2 depletion in comparison to control cells from the data used for D. F) Average NuMA intensity at the cortex measured from regions of interest (ROIs) drawn around the cortex in metaphase cells - 30 cells (control), 20 cells (LIC1 depleted) and 20 cells (LIC2

depleted) over a total of 3 experiments. Error bars are mean +/- SEM from 3 independent experiments.

Supplementary figure S6

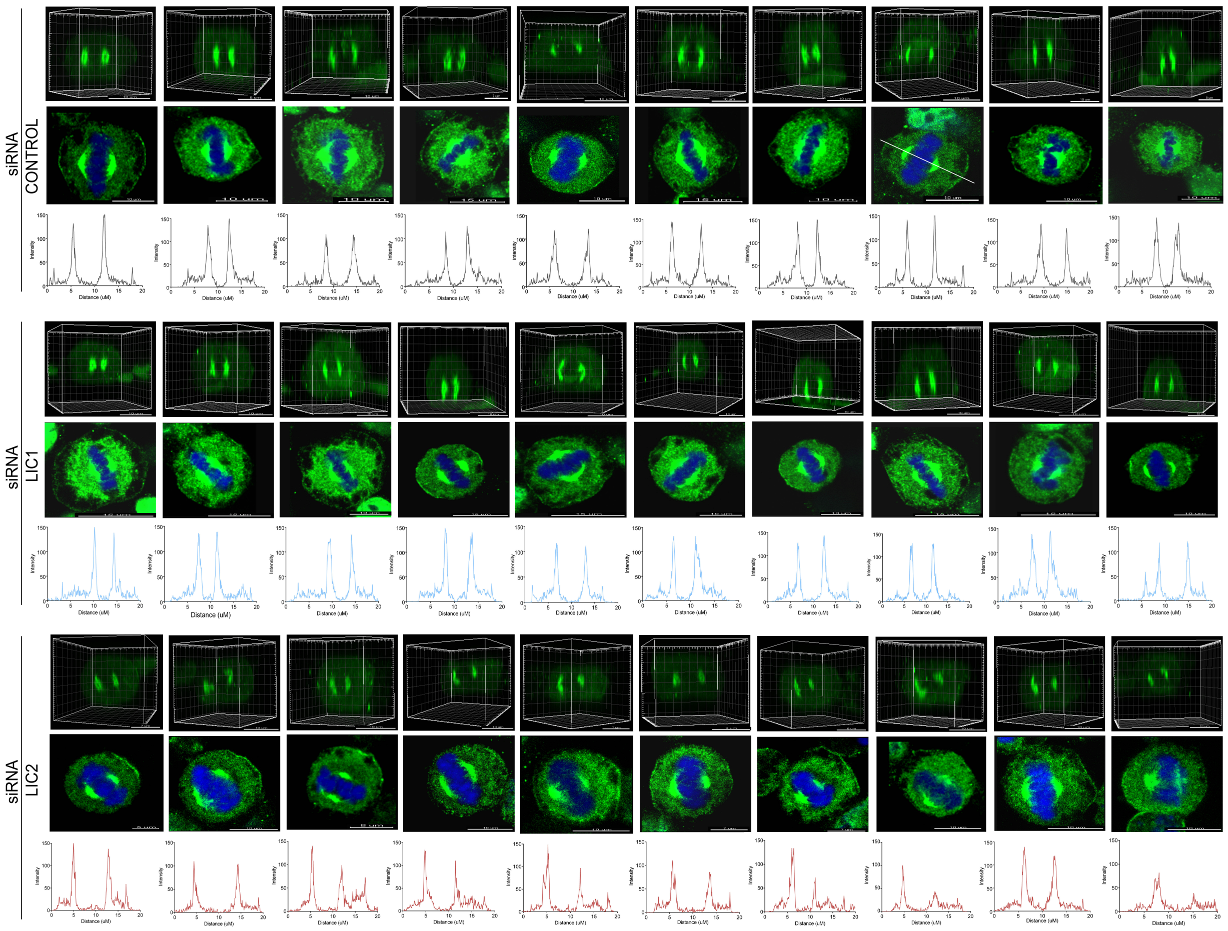

Supplementary figure S6: Additional figure showing representative images (ten) of 3D reconstructions with 2D line scan measurements and graphical representation of the same line scans of NuMA intensity along the spindle axis. *Cells have been shown with brightness and contrast adjusted to show the cortex.*

Supplementary figure S7

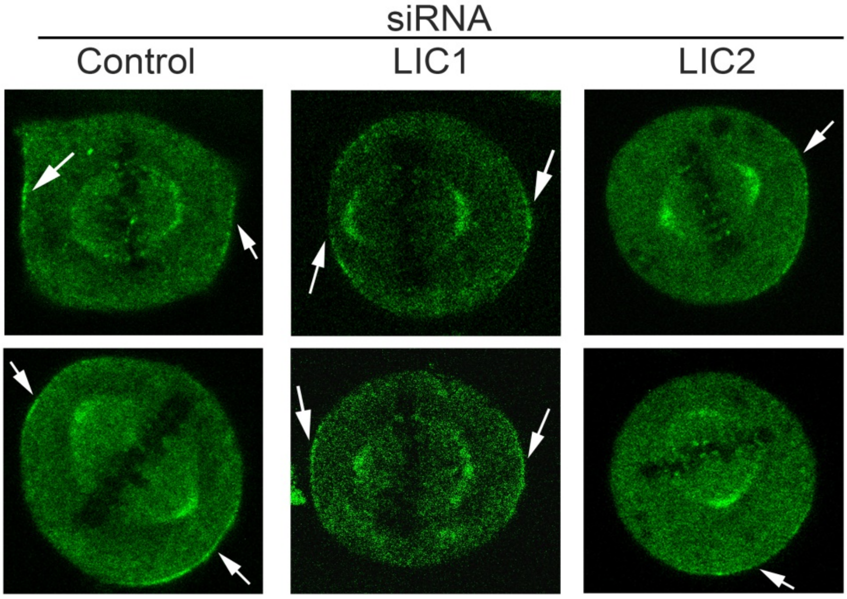

Supplementary figure S7: Additional figure showing extra representative images in support of figure 5A.

Still images from time-lapse movies of HeLa cells expressing multifunctional GFP-tagged IC74 (mfGFP-IC74). Arrows indicate cortical regions proximal to the spindle poles.

Supplementary figure S8

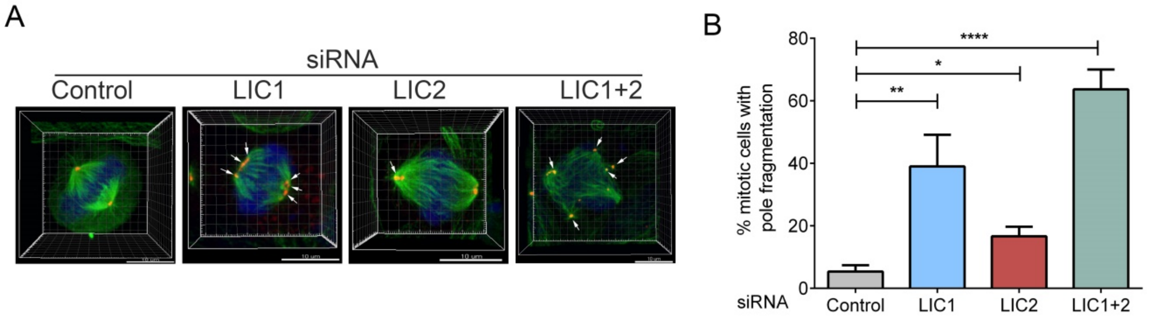

Supplementary figure S8*: *LIC1 plays a dominant role in preventing spindle pole fragmentation

A) Representative images showing fragmented pole defects upon LIC1 and LIC2 depletion, as compared to control bipolar HeLa cells. B) Quantification of the fragmented pole defects. Cells were stained for microtubules (α-tubulin, green), spindle poles (gamma tubulin, red) and chromatin (DAPI, blue). Y-axis depicts the fraction of mitotic cells with fragmented poles. Error bars are mean +/- SD from 3 independent experiments, at least 20 metaphase cells counted per experiment.

Supplementary figure S9

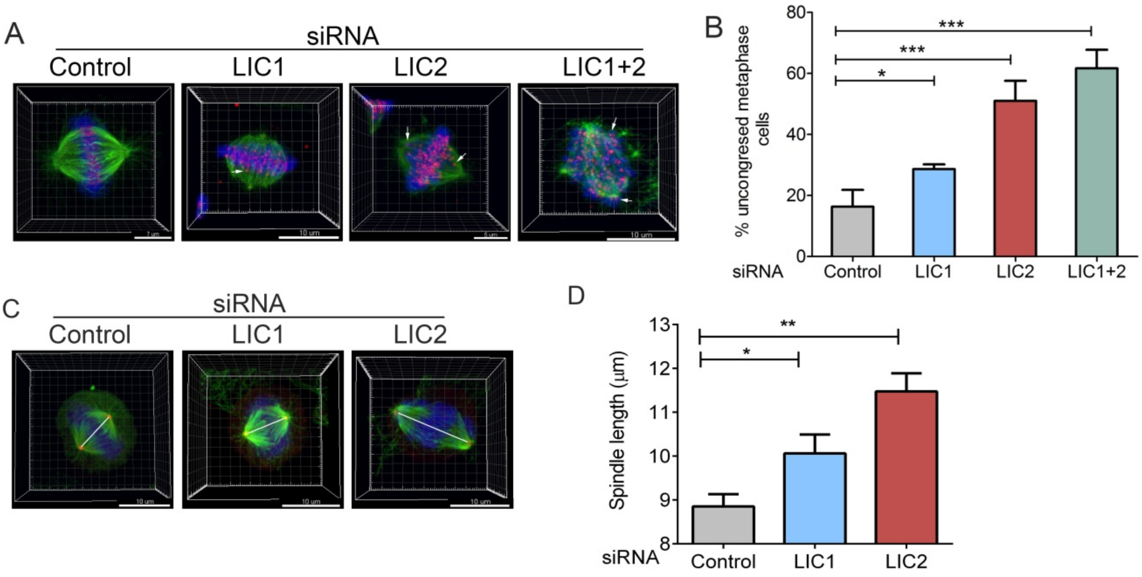

***Supplementary figure S9*: LIC2 is required for chromosome congression and for regulating mitotic spindle length**

A) Mitotically arrested HeLa cells stained for microtubules (green), kinetochores (red) and chromatin (blue). White arrows indicate uncongressed kinetochores. B) Fraction of metaphase cells with uncongressed chromosomes (3 experiments, n = at least 20 metaphase arrested HeLa cells per experiment. C) Spindle length (white line) measured from metaphase arrested HeLa cells stained as in A, except red = centrosome (gamma tubulin). D) Average mitotic spindle length (y-axis) +/- SD, n = 3 experiments, at least 20 HeLa metaphase arrested cells per experiment.

Supplementary figure S10

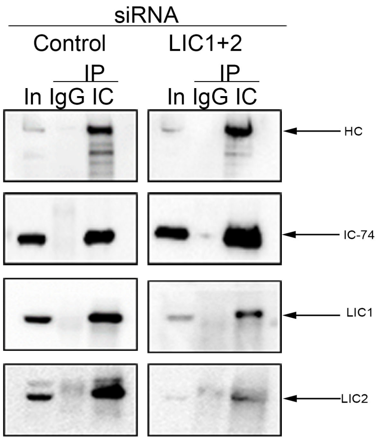

Supplementary figure S10: Dynein retains its integrity upon depletion of the LICs. Immunoprecipitation with IC74 co-precipitates HC irrespective of LIC depletion. In= Input, IP = immunoprecipitation.

Supplementary movie legends

Supplementary movies 1, 2, 3and 4: Fluorescence time-lapse movies of control, LIC1-depleted, LIC2-depleted and LIC1 + LIC2 co-depleted live H2B-mCherry GFP-α-tubulin HeLa cells respectively showing the duration of mitosis.

Supplementary movies 5, 6, 7, and 8: Fluorescence time-lapse movies of H2B-mCherry GFP-α-tubulin HeLa cells showing the duration of mitosis in control, 30 μM dynein inhibitor, control and 50 μM dynein inhibitor respectively.

Supplementary movies 9, 10: Fluorescence time-lapse movie (3D reconstruction) of control and LIC2-depleted live H2B-mCherry GFP-α-tubulin HeLa cells showing the spindle oriented parallel to the substratum or severely misoriented respectively.

Supplementary movies 11, 12: Bright field time-lapse movie of control and LIC2 depleted HeLa cells showing even/ uneven flattening respectively of both daughter cells to the substratum during cytokinesis.

Supplementary movies 13, 14, 15: Videos of control, LIC1 siRNA treated and LIC2 siRNA treated live H2B-mCherry GFP-α-tubulin HeLa cells after treatment with MG132 (10 μM) for 4 hours. Only LIC2-depleted cells show drastic spindle mis-orientation.

Supplementary movie 16, 17, 18: Video of control, LIC1 and LIC2 depleted HeLa cells stably expressing multifunctional GFP-tagged IC74, showing cortically localized dynein and loss of cortical dynein respectively.

Supplementary movies 19, 20, 21: Videos of control, LIC1 siRNA treated and LIC2 siRNA treated live H2B-mCherry GFP-α-tubulin HeLa cells after treatment with MG132 (10 μM) for 4 hours. Only LIC1-depleted cells show drastic spindle pole fragmentation.
